# Cytotoxic Stilbenes and Canthinone Alkaloids from *Brucea antidysenterica* (Simaroubaceae)

**DOI:** 10.3390/molecules24234412

**Published:** 2019-12-03

**Authors:** Yves Salomon Makong, Gervais Mouthé Happi, Judith Liliane Djouaka Bavoua, Jean Duplex Wansi, Lutfun Nahar, Alain François Kamdem Waffo, Claire Martin, Norbert Sewald, Satyajit Dey Sarker

**Affiliations:** 1 Department of Chemistry, Faculty of Science, University of Douala, P.O. Box 24157 Douala, Cameroon; y.makong@yahoo.com (Y.S.M.); dbjl19@yahoo.fr (J.L.D.B.); akamdemfr@yahoo.fr (A.F.K.W.); 2 Organic and Bioorganic Chemistry, Department of Chemistry, Bielefeld University, 33501 Bielefeld, Germany; gervais20022003@yahoo.fr; 3 Laboratory of Growth Regulators, Institute of Experimental Botany ASCR & Palacký University, Šlechtitelů 27, 78371 Olomouc, Czech Republic; L.Nahar@ljmu.ac.uk; 4 Centre for Natural Products Discovery (CNPD), School of Pharmacy and Biomolecular Sciences, Liverpool John Moores University, James Parsons Building, Byrom Street, Liverpool L3 3AF, UK; S.Sarker@ljmu.ac.uk; 5 Department of Biological Sciences, School of Science and the Environment, University of Worcester, Henwick Grove, Worcester WR2 6AJ, UK; c.martin@worc.ac.uk

**Keywords:** *Brucea antidysenterica*, Simaroubaceae, bruceanoside A, bruceacanthinones A–B, cytotoxicity

## Abstract

A phytochemical study of the root and bark of *Brucea antidysenterica* J. F. Mill. (Simaroubaceae) afforded three new compounds, including a stilbene glycoside bruceanoside A (**1**), and two canthinone alkaloids bruceacanthinones A (**3**) and B (**4**), along with ten known secondary metabolites, rhaponticin (**2**), 1,11-dimethoxycanthin-6-one (**5**), canthin-6-one (**6**), 1-methoxycanthin-6-one (**7**), 2-methoxycanthin-6-one (**8**), 2-hydroxy-1,11-dimethoxycanthin-6-one (**9**), *β*-carboline-1-propionic acid (**10**), cleomiscosin C (**11**), cleomiscosin A (**12**), and hydnocarpin (**13**). The structures of all the compounds were determined using spectrometric and spectroscopic methods including 1D and 2D NMR, and HRSEIMS. The identities of the known compounds were further confirmed by comparison of their data with those reported in the literature. The root and bark methanolic extracts, the dichloromethane and ethyl acetate soluble fractions, and the isolated compounds (**3**–**13**), were assessed for their cytotoxicity against the cancer cell lines A-549, MCF-7, and PC-3. The results suggested that compounds in the extracts might possess a synergic action in their cytotoxicity.

## 1. Introduction

Plants from the *Brucea* genus (Simaroubaceae) have been extensively investigated for their chemical constituents and pharmacological properties since 1900 [1]. Among the numerous metabolites reported so far, quassinoids, also called nigakilactones and canthinone alkaloids, are the most important class of compounds [1], with demonstrable biological activities, including anti-amoebic [2], antitumour [3,4,5], antiplasmodial [6,7], and antituberculosis [8] effects. As the name indicates, *Brucea antidysenterica* J. F. Mill. is used in traditional medicine for the treatment of dysentery and also against malaria, diarrhoea, and stomach aches [9,10]. In Ethiopia, the plant is also used for the treatment of tumors, and a follow-up investigation revealed that the compound has promising activity in vitro against several lymphoma, leukemia, and myeloma cell lines [11]. In addition, the 80% methanolic extract from the leaf was assayed for antioxidant activity, showing significant inhibition of 87.57% for the water soluble fraction as measured by DPPH assay, and 72.57 mg AAE/g as measured by FRAP assay [12]. Moreover, 50 mg/mL of the aqueous leaf extract gave a better wound healing effect than the control group when applied topically on a mouse model [13]. The aqueous, methanolic, and chloroform crude extracts of the seeds delivered significant *Plasmodium berghei* suppression in 6–8 week old Swiss albino mice of both sexes when given at 600 mg/kg body weight, while the acute toxicity test at a single dose of 2000 mg/kg body weight led to the death of all mice within 24 h for each of above extracts [14]. It should be noted that the first pure compound reported from the plant, the triterpene quassinoid bruceantin, was already isolated in 1973 from the alcoholic stem bark extract and shown in the follow-up to possess activities related to cell apoptosis and proliferation, angiogenesis, inflammation, and metabolic diseases [11,15,16]. Recently, we identified bruceadysentoside A, a pregnane glycoside, from the leaves of *B. antidysenterica*, in addition to seventeen other compounds, including hydnocarpin, which exhibited the strongest antiproliferative activity with IC_50_ values of ≈100 µM and ≈200 µM against PC-3 and HT-29, respectively [17]. In continuation of our program to explore the phytochemicals of *B. antidysenterica* and their cytotoxicity, we investigated the bark and root of this plant. In this report, we describe the isolation and structural elucidation of one new stilbene (**1**) and two new canthinone alkaloids (**3** and **4**) and their cytotoxic activities.

## 2. Results

**Figure 1:**
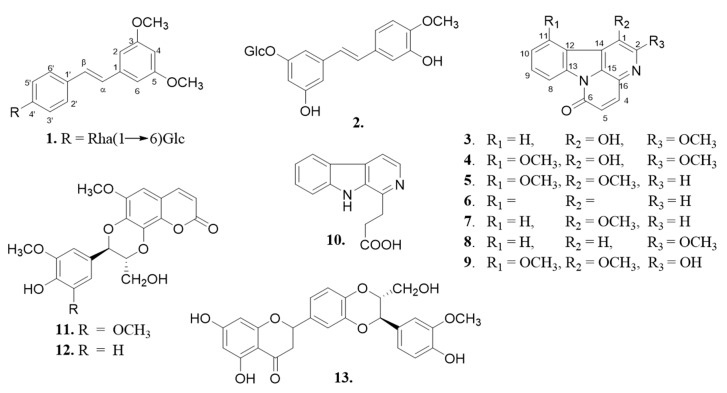
Structures of compounds (**1**–**13**) isolated from *Brucea antidysenterica*.

The bark and root of *B. antidysenterica* were separately extracted with MeOH. A small amount of each extract was stored for their in vitro-(4,5-dimethylthiazol-2-yl)-2,5-diphenyltetrazolium bromide (MTT) cytotoxicity/viability assay on A-549, MCF-7, and PC-3 cell lines. Based on their similar Thin-Layer Chromatography (TLC) profiles both bark and root extracts were combined and partitioned with dichloromethane and ethyl acetate to afford two major fractions. Subsequent purification of these fractions led to the isolation of three new compounds, including one new stilbene glycoside (**1**) and two new canthinone alkaloids (**3** and **4**). The comparison of our data with those reported in the literature allowed identification of the known compounds, such as rhaponticin (**2**) [18], 1,11-dimethoxycanthin-6-one (**5**) [19], canthin-6-one (**6**) [19], 1-methoxycanthin-6-one (**7**) [20], 2-methoxycanthin-6-one (**8**) [21], 2-hydroxy-1,11-diméthoxycanthin-6-one (**9**) [22], *β*-carboline-1-propionic acid (**10**) [23], cleomiscosin C (**11**) [24], cleomiscosin A (**12**) [25], and hydnocarpin (**13**) [26] (Figure 1).

**Figure 2:**
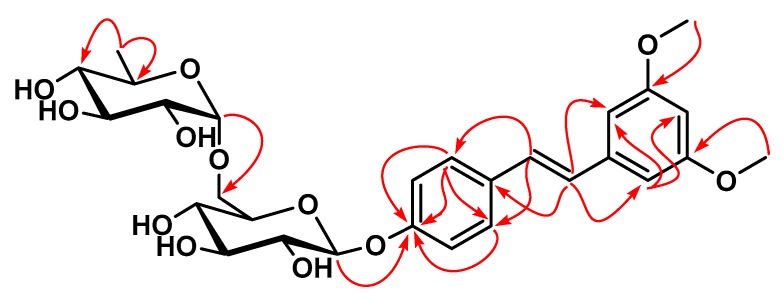
Select, key HMBC correlations of **1**.

Compound **1** was obtained as a white amorphous powder. Its molecular formula C_29_H_39_O_11_ was deduced from the ESIMS (Figures S1 and S2) and from the (+)-HR-ESIMS showing the pseudo-molecular ion peak at *m*/*z* 563.2488 [M + H]^+^ (calculated for C_29_H_39_O_11_, 563.2492). The ^1^H NMR spectrum (Table 1, Figure S3) showed characteristic signals of two aromatic methoxyl (*δ* 3.80, 6H), two *trans*-olefinic protons (*δ* 6.99 and 7.20, d, *J =* 16.4 Hz, 1H each), and the occurrence of two independent aromatic rings, each carrying three protons organized in two different spin systems: The first ring (A) showed the AMX-type signals (*δ* 6.78 and 6.82, brs, 1H each; 6.52, t, *J* = 2.2 Hz, 1H) and were attributed to the protons of a 1,3,5-trisubstituted aromatic ring, while the second ring (B) exhibited ^1^H signals of an A_2_B_2_-coupled spin system (*δ* 6.95 and 7.58, d, *J* = 8.8 Hz, 2H each) which were assignable to the protons of a 1,4-disubstituted aromatic ring. Furthermore, the presence of two anomeric proton signals was depicted at *δ* 4.89 (1H, d, *J* = 7.6 Hz) and 4.56 (1H, d, *J* = 1.7 Hz), and a methyl doublet of rhamnose was at *δ* 1.11 (3H, d, *J* = 6.1 Hz). These three ^1^H signals coupled to the HSQC and DEPT experiments allowed us to assign their ^13^C resonances at *δ* 101.0, 101.0, and 18.3, respectively (Figures S4–S6). All these findings indicated that compound **1** was a stilbene glycoside with two sugar moieties; one of them might be rhamnose [27]. Careful analysis of the HMBC spectrum (Figure 2 and Figure S7) of compound **1** showed cross-peak correlations between the proton signal at *δ* 6.99 (H-*α*) and the carbon signals of the ring A appearing at *δ* 129.9 (C-1), 107.6 (C-6), and 105 (C-2), while the proton signal at *δ* 7.20 (H-*β*) showed correlations with the carbon signals involved in the ring B and appearing at *δ* 139.7 (C-1′) and 128.4 (C-2′/C-6′), confirming that rings A and B are linked through the *trans*-orientated bond C*_α_*–C*_β_* characteristic of stilbene. Furthermore, the two methoxy groups were connected at C-3 and C-5 in ring A according to the correlations observed between the proton signals at *δ* (3.80, s, 6H) and the carbon signals at *δ* 159.3 (C-3) and 160.2 (C-5). Therefore, the aglycone was characterized to be pterostilbene, and the two remaining sugar moieties might be connected at the only position C-4′ (*δ*_C_159.8) in cycle B suggesting that **1** is a pterostilbene glycoside [28]. This partial conclusion was further supported by the mass spectra in positive and negative ionization mode (Figures S1 and S2). Briefly, the (–)-HR-ESIMS showed a peak at 255.106 corresponding to the fragment [M − rha − glc]^−^; while the (+)-HR-ESIMS exhibited signal at 441.156 attributed to the fragment [M + Na − rha]^+^. Both observations indicated that the two sugar units are linked to each other and connected to the aglycone pterostilbene via the glucose subunit. Further proof was obtained from the long-range correlation between the anomeric proton of glc at *δ* 4.89 (d, *J* = 7.6 Hz, glc H-1) and the carbon signal at *δ* 159.8 (C-4′). The linkage of the two sugar moieties was established from the HMBC correlations between *δ* 4.56 (1H, d, *J* = 1.6 Hz, ara H-1) and *δ* 66.8 (glc C-6) (Figure 2). The anomeric proton of the glucose was assigned with *β*-configuration based on its ^3^*J*_H-1,H-2_ coupling constant of 7.6 Hz (Table 1), while the anomeric proton of rhamnose was assigned with *α*-configuration due to the characteristic ^13^C chemical shift of C-5 (rha) at *δ* 68.8 (Table 1) [29]. Based on all these above evidence, the structure of compound **1** was assigned to be pterostilbene 3-*O*-*α*-rhamnopyranosyl(1→6)-*β*-glucopyranoside and given the trivial name, bruceanoside A.

**Figure 3:**
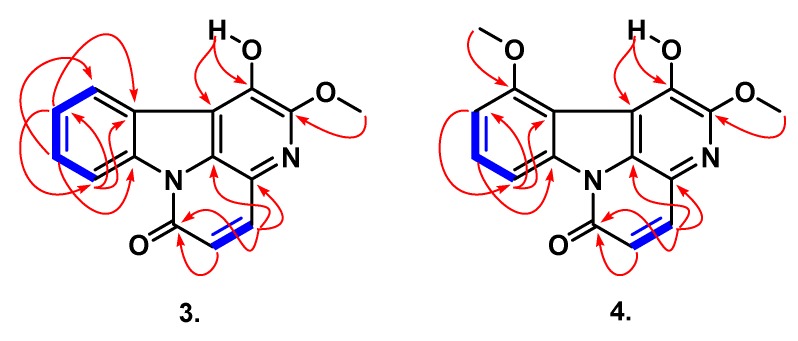
Select, key COSY (blue) and HMBC (red) correlations of **3** and **4**.

Compound **3**, named bruceacanthinone A, was obtained as an amorphous yellow solid. Its molecular formula was assigned as C_15_H_10_N_2_O_3_ with help of the ESIMS (Figure S10) and the (+)-HR-ESIMS (*m*/*z* 289.0570 [M + Na]^+^, calculated for C_15_H_10_N_2_O_3_Na, 289.0589) counting for 12 double-bond equivalents. The Dragendorff’s test was positive, suggesting that **3** was an alkaloid. On its ^1^H NMR spectrum (Table 2, Figure S11), the signals of two *cis*-coupled protons [*δ* 8.08 and 6.90 (d, *J* = 9.7 Hz, 1H each)] were observed, which are characteristic of the lactam ring of canthin-6-one [30]. Furthermore, there were signals corresponding to one methoxyl at *δ* 4.29 (3H, s), one hydroxyl at *δ* 8.52 (1H, s), and signals of a four-spin ABMX-type system supported by the COSY correlations (Figure 3) observed between the four proton signals at *δ* 8.70 (1H, dd, *J* = 1.2 and 7.8 Hz), 8.26 (1H, dd, *J* = 1.0 and 7.6 Hz), 7.70 (1H, td, *J* = 1.0, 7.6, and 7.8 Hz), and 7.58 (1H, td, *J* = 1.2, 7.6, and 7.8 Hz). The ^13^C NMR spectrum for **3** (Table 2, Figure S12)) confirmed 16 carbon signals which were clearly sorted by and HSQC (Figure S13) experiments into one methoxyl (*δ* 57.2), one ketone carbonyl characteristic of conjugated lactam (*δ* 159.6), six methines, and seven quaternary carbons. All these data were similar to those of huberine, a canthin-6-one alkaloid recently reported from the stem bark of *Picrolemma huberi* [31]. The comparison of their NMR data suggested that, besides some changes in chemical shifts, which could be explained by the solvent effect during the analysis, the main and more relevant change is the presence of ^1^H and ^13^C signals of two methoxyl groups in huberine rather than one in compound **3**. This partial conclusion could be further confirmed by the difference of 14 mass units between the two compounds. The HMBC spectrum of **3** (Figure 3 and Figure S14) showed the same cross-peaks observed in huberine, indicating that the two compounds share the same core structure [31]. Furthermore, the HMBC experiment allowed the assignment of the methoxyl unit in C-2 and the hydroxyl group in C-1 according to the cross-peaks observed between *δ* 4.29 (OCH_3_) and *δ* 159.6 (C-2), and between *δ* 8.52 (OH) and *δ* 159.6 (C-2), 140.0 (C-1), and 119.2 (C-14). On the basis of all the evidence obtained, the structure of **3** was deduced as 1-hydroxy-2-methoxycanthin-6-one.

Compound **4** was isolated as an amorphous yellow solid and its molecular formula was deduced from its ESIMS (Figure S16) and HR-ESIMS as C_16_H_12_N_2_O_4_ (*m*/*z* 297.0855, calculated for C_16_H_13_N_2_O_4_, 298.0875), exhibiting 30 amu more than that of **3**. We found that compounds **3** and **4** are structurally related according to their close ^1^H and ^13^C NMR data (Table 2, Figures S17 and S18). The two main differences were the presence of an additional methoxyl group in compound **3** (*δ* 4.28, 57.8) and the occurrence of a three-spin ABC-system rather of the four-spin ABMX-system previously observed in **3**. All these findings suggested that compound **4** is a derivative of **3** in which a methoxyl group is attached as substituent on the ring A. Therefore, the placement of this additional methoxyl group was easily done with the HMBC experiment (Figure 3 and Figure S19) which exhibited a cross-peak of correlation between *δ* 4.28 (3H, s) and *δ* 160.0 (C-11). The structure of bruceacanthinone B (**4**) was finally determined as 2,11-dimethoxy-1-hydroxycanthin-6-one.

The root and bark crude extracts, their dichloromethane and ethyl acetate soluble main fractions, and the compounds **3**–**13**, were assessed for their cytotoxic potency by MTT assay against three human cancer cell lines A-549, MCF-7, and PC-3 using etoposide as the positive control (3.5 ± 0.5, 10.2 ± 1.2, and 7.9 ± 1.1 µg/mL, respectively for A-549, MCF-7, and PC-3 see Table 3). The crude extracts and the fractions displayed cytotoxicity with IC_50_ values ranging from 50.0 ± 5.2 to 80.5 ± 1.8 µg/mL. However, the potencies of isolated compounds **3**–**13** were indicated by IC_50_ values more than the double of those of crude extracts (Table 3). This observation supported that the isolated compounds were less cytotoxic in single assay compared to the crude extracts and fractions. The results suggested that compounds from *B. antidysenterica* root and bark extracts might have a synergic effect in cytotoxicity assay.

## 3. Materials and Methods

### 3.1. General Experimental Procedures

Chromatographic solvents were purchased from Fisher Scientific, UK, and used without further purification. Optical rotations were measured in methanol on a JASCO DIP-360 digital polarimeter using a 10 cm cell. Ultraviolet spectra were recorded on a Hitachi UV 3200 spectrophotometer in MeOH. Infrared spectra were recorded on a JASCO 302-A spectrophotometer. ESIMS was recorded on a Finnigan LCQ with a Rheos 4000 quaternary pump (Flux Instrument). EIMS were recorded on a Varian MAT 311A spectrometer (70 eV). EIMS was performed on a JEOL HX 110 mass spectrometer. The ^1^H and ^13^C NMR spectra were recorded at 600 and 150 MHz, respectively, on a Bruker AMX 600 NMR spectrometer. Chemical shifts are reported in *δ* (ppm). Column chromatography was carried out on silica gel (70–230 mesh, Merck) and flash silica gel (230–400 mesh, Merck). TLC was performed on Merck precoated silica gel 60 F_254_ aluminum foil, using ceric sulfate spray reagent for visualization. The purity of compounds was investigated by HPLC. The degree of purity of the positive control compound was ≥98%, while that of the isolated compounds was ≥95%. All reagents used were analytical grade.

### 3.2. Plant Material

The bark and root of *B. antidysenterica* J. F. Mill. were collected in June 2013 at Bakouok (Bamenda) in the Northwest Region of Cameroon and were authenticated by Nana Victor, a botanist at the National Herbarium of Cameroon where a voucher specimen was deposited under the registration number 54,605 HNC.

### 3.3. Extraction and Isolation

The air-dried and powdered bark (2.5 kg) and root (1.2 kg) of *B. antidysenterica* were separately extracted twice at room temperature with MeOH for 48 h and 24 h, respectively. The solvent was removed by evaporation under reduced pressure to obtain the crude extracts from bark (85.6 g) and root (38.7 g). A small amount (≈5 g) of each extract was kept for cytotoxicity assay against three human cancer cell lines A-549, MCF-7, and PC-3. The two crude extracts were combined according to their very similar compositions observed on a TLC plate. The mixed crude extract (114.3 g) was dissolved in water and partitioned with *n*-hexane, dichloromethane (DCM), and ethyl acetate (EtOAc) to afford three main fractions A (12.2 g), B (23.6 g), and C (41.7 g), respectively. Fraction A was mainly oils and fats; the remaining water-soluble fraction was dried and kept for further analysis while the fractions B and C were purified to yield the thirteen compounds identified in this report. More closely, the DCM soluble fraction B was subjected to silica gel column chromatography using a gradient of EtOAc in *n*-hexane to obtain a total of 73 fractions which were combined based on their TLC profiles into four main subfractions B1–B4. Repeated silica gel column chromatography of the subfractions B1 (7.3 g) and B2 (5.8 g) separately eluted with a gradient of DCM in *n*-hexane yielded compounds **6** (10.5 mg) and **7** (15.5 mg) from B1, while compound **8** (8.7 mg) was obtained from B2. Similarly, the subfractions B3 (6.2 g) was further purified on silica gel column chromatography with DCM-EtOAc gradient to give compounds **3** (9.3 mg), **4** (6.4 mg), and **5** (4.2 mg). Additionally, the EtOAc soluble fraction C was chromatographed on silica gel with a DCM-EtOAc gradient followed by a gradient of MeOH in EtOAc. As a result, 72 fractions of ca. 100 mL each were collected and combined into the subfractions C1–C3 on the basis of TLC. Purification of C1 (10.8 g) over a silica gel column chromatography with the isocratic system DCM-EtOAc (1:2) gave compounds **9** (12.0 mg), **10** (14.0 mg), and **12** (11.8 mg). In the same way, the subfractions C2 (12.4 g) and C3 (14.3 g) were separately chromatographed on silica gel eluted with the isocratic system of 25% DCM in EtOAc to yield compounds **11** (12.3 mg) and **13** (8.7 mg) from C2, and compounds **1** (9.6 mg) and **2** (5.6 mg) from C2.

#### 3.3.1. Bruceanoside A (**1**)

White amorphous powder; mp −80.2° (ca. 0.4 acetone); R_f_ = 0.25 (CHCl_3_/MeOH, 4/1); IR (KBr) ν_max_ 3348, 2940, 2892, 1615, 1593, 1350, 1265, 1255, 890, 750 cm^−1^; UV (MeOH) λ_max_ (log ε): 290 (4.25), 265 (4.15) nm; ^1^H NMR (600 MHz, DMSO-*d_6_*) and ^13^C NMR (150 MHz, DMSO-*d_6_*) data, see Table 1; HRESIMS *m*/*z* 563.2488 [M + H]^+^ (calculated for C_29_H_39_O_12_, 563.2492).

#### 3.3.2. Bruceacanthinone A (**3**)

Yellowish powder; R_f_ = 0.45 (CHCl_3_/MeOH, 9.5/0.5); IR (KBr) ν_max_ 3375, 2950, 2350, 1690, 1600, 1435, 1142, 865, 765, 720 cm^−1^; UV (MeOH) λ_max_ (log ε): 398 (3.95), 380 (3.90), 365 (3.60), 329 (4.10), 250 (4.70); ^1^H NMR (600 MHz, DMSO-*d_6_*) and ^13^C NMR (150 MHz, DMSO-*d_6_*) data, see Table 2; HRESIMS *m*/*z* 289.0589 [M + Na]^+^ (calculated for C_15_H_10_N_2_O_3_Na, 289.0570).

#### 3.3.3. Bruceacanthinone B (**4**)

Yellowish powder; R_f_ = 0.40 (CHCl_3_/MeOH, 9.5/0.5); IR (KBr) ν_max_ 3370, 2945, 2350, 1700, 1650, 1440, 1143, 870, 766, 722 cm^−1^; UV (MeOH) λ_max_ (log ε): 395 (3.90), 376 (3.92), 367 (3.61), 329 (4.15), 255 (4.65); ^1^H NMR (600 MHz, DMSO-*d_6_*) and ^13^C NMR (150 MHz, DMSO-*d_6_*) data, see Table 2; HRESIMS *m*/*z* 297.0855 [M + H]+ (calculated for C_16_H_13_N_2_O_4_, 298.0875).

### 3.4. Cell Lines, Cell Cultures, and the MTT Assay

The potential cytotoxicity of *Brucea antidysenterica* bark and root extracts, their VLC fractions, and some isolated compounds (**3**–**10**) were studied against cancer cell lines A-549 (human lung adenocarcinoma), MCF-7 (human breast adenocarcinoma), and PC-3 (human prostate adenocarcinoma) using the MTT assay [32,33]. Methods used for toxicity assays have been reported by us previously in detail [34] and are presented here again. All cell lines were cultured in RPMI-1640 medium supplemented with 10% fetal bovine serum and 1% antibiotic-antimycotic solution (100×). The cells were cultured at 37 °C in 95% humidity and 5% CO_2_. For the MTT assays, the cells were washed using phosphate-buffered saline and harvested by trypsinization. Cells were then seeded into 24 well plates at a density of 1.2 × 10^4^ cells/well in a working volume of 1 mL/well and allowed to grow for 24 h before the commencement of each experiment. The cells were then treated for 24 h with different concentrations of test samples (crude extracts and fractions at 0, 0.8, 4, 20, 100, and 500 μg/mL and the compounds at 0, 0.4, 2, 10, 50, 75, and 100 μg/mL). Dilutions of stock solutions were made in culture media, yielding final sample concentrations and a final dimethyl sulfoxide (DMSO) concentration of 0.1%, including the control. Each sample concentration was used to treat four wells of cells in each 24 well plate. After the 24 h treatment period, the toxicity of the samples on each cell line was quantified. To achieve this, the medium in each well was replaced by MTT solution (500 μg/mL in medium) and incubated for 2 h. Viability of cells was assessed by their ability to reduce the yellow dye MTT to a blue formazan product [35]. The MTT reagent was removed, the formazan crystals were dissolved in isopropanol, and the absorbance at 560 nm determined using a microplate reader (CLARIO Star Microplate Reader, BMG Labtech, UK). The average absorbance value obtained from zero treatment control (0.1% DMSO) wells was arbitrarily set at 100% for each plate, and the absorbance value for the average of wells of cells treated with each test sample concentration was expressed as a percentage of this control. Each assay was performed on a minimum of three separate occasions, and the IC_50_ values for each sample on each cell line were calculated using Microsoft Excel version 2013. The anti-lung cancer semisynthetic glycoside drug etoposide was used as a positive control.

### 3.5. Statistical Analysis

All experiments were carried out in triplicate on separate occasions. Data were expressed as means ± standard errors of the means. The graphs were plotted using nonlinear regression with the use of Microsoft Excel version 2013.

## 4. Conclusions

The chemical investigation of the root and bark of *Brucea antidysenterica* afforded three new compounds, including one stilbene glycoside bruceanoside A (**1**), and two canthinone alkaloids bruceacanthinones, A (**3**) and B (**4**), along with ten known secondary metabolites sorted into six canthinone alkaloid derivatives (**5**–**10**), two coumarin derivatives (**11** and **12**), one stilbene (**2**), and one flavonoid (**13**). As expected from the genus *Brucea*, canthinone alkaloids were the most abundant class of metabolites isolated during this study and might be classified as chemo markers of *Brucea* genus. Furthermore, the cytotoxicity of the root and bark methanolic extracts, the dichloromethane and ethyl acetate soluble fractions, and the isolated compounds (**3**–**13**), were assessed against the cancer cell lines A-549, MCF-7, and PC-3. The results displayed decreasing of IC_50_ values from crude extracts to the isolated compounds, and might suggest a synergistic action of compounds in their cytotoxicity. Since the plant is used in traditional medicine, its extract might be considered an important reservoir of cytotoxic compounds for the treatment of tumors. However, additional in vivo toxicity evaluation of extracts, antiplasmodial or antibacterial activities of extracts and compounds, and chemical investigation of other parts of the plants—leaves and fruits for instance—in addition to supporting the use of this plant in folk medicine, will help to obtain further information to enrich the pharmacology and chemistry of the genus *Brucea*.

## Figures and Tables

**Table 1:** ^1^H (600 MHz) and ^13^C (150 MHz) NMR assignments of **1** in DMSO-*d_6_*.

No	1	
	*δ*_C_	*δ*_H_ (mult., *J* in Hz)
***α***	126.4	6.99 (1H, d, 16.4)
***β***	129.1	7.20 (1H, d, 16.4)
**1**	129.9	–
**2**	105.6	6.82 (1H, brs)
**3**	159.3	–
**4**	101.9	6.52 (1H, t, 2.2)
**5**	160.2	–
**6**	107.6	6.78 (1H, brs)
**1′**	139.7	–
**2′/6′**	128.4	7.58 (2H, d, 8.8)
**3′/5′**	114.6	6.95 (2H, d, 8.8)
**4′**	159.8	–
**OCH_3_-3/5**	55.6	3.80 (6H, s)
sugar moieties		
**glc-1**	101.0	4.89 (1H, d, 7.6)
**2**	73.7	3.19 (1H, m)
**3**	75.8	3.52 (1H. m)
**4**	70.8	3.65 (1H, m)
**5**	76.9	3.30 (1H, m)
**6**	66.8	3.41 (1H, m) 3.87 (1H, m)
**rha-1**	101.0	4.56 (1H, d, 1.6)
**2**	71.2	3.42 (1H, m)
**3**	70.3	3.46 (1H, m)
**4**	72.5	3.18 (1H, m)
**5**	68.8	3.43 (1H, m)
**6**	18.3	1.11 (3H, d, 6.1)

**Table 2:** ^1^H (600 MHz) and ^13^C (150 MHz) NMR assignments of **3** and **4** in DMSO-*d_6_*.

No	3		4	
	*δ*_C_	*δ*_H_ (mult., *J* in Hz)	*δ*_C_	*δ*_H_ (mult., *J* in Hz)
**1**	140.0	–	140.4	–
**2**	152.6	–	151.5	–
**4**	139.0	8.08 (1H, d, 9.7)	139.9	8.04 (1H, d, 9.8)
**5**	127.3	6.90 (1H, d, 9.7)	125.9	6.90 (1H, d, 9.8)
**6**	159.6	–	160.0	–
**8**	117.1	8.70 (1H, dd, 1.2, 7.8)	112.9	8.40 (1H, dd, 0.8, 8.2)
**9**	130.7	7.70 (1H, td, 1.0, 7.6, 7.8)	132.3	7.65 (1H, t, 8.2)
**10**	127.5	7.58 (1H, td, 1.2, 7.6, 7.8)	108.0	7.01 (1H, dd, 0.8, 8.2)
**11**	125.0	8.26 (1H, dd, 1.0, 7.6)	156.1	–
**12**	123.1	–	126.8	–
**13**	136.4	–	139.9	–
**14**	130.7	–	132.9	–
**15**	128.7	–	132.3	–
**16**	126.1	–	126.8	–
**OMe-2**	57.2	4.29 (3H, s)	56.3	4.09 (3H, s)
**OMe-11**	–	–	57.8	4.28 (3H, s)
**OH-1**	–	8.52 (1H, s)	–	8.50 (1H, s)

**Table 3:** Cytotoxicity of extracts, fractions, and isolated compounds from *Brucea antidysenterica.*

Samples	IC_50_ in μg/mL		
	A-549	MCF-7	PC-3
**Root extract**	75.2 ± 3.2	80.5 ± 1.8	77.7 ± 2.5
**Bark extract**	65.1 ± 1.5	72.3 ± 3.5	70.2 ± 4.2
**DCM**	50.0 ± 5.2	55.1 ± 4.2	57.0 ± 5.3
**EA**	61.5 ± 3.2	58.1 ± 1.9	55.8 ± 2.4
**3**	>250	>250	195.5 ± 9.5
**4**	150.3 ± 8.5	157.5 ± 7.5	160.5 ± 5.5
**5**	>250	>250	>250
**6**	175.6 ± 6.1	170.3 ± 5.8	177.3 ± 5.9
**7**	125.8 ± 7.2	152.1 ± 11.2	155.1 ± 5.3
**8**	121.5 ± 8.2	126.2 ± 5.5	130.9 ± 7.4
**9**	138.1 ± 9.7	145.2 ± 9.9	151.5 ± 8.3
**10**	>250	>250	>250
**11**	>250	>250	>250
**12**	130.8 ± 7.4	132.1 ± 11.2	130.1 ± 5.3
**13**	>250	>250	>250
**Etoposide**	3.5 ± 0.5	10.2 ± 1.2	7.9 ± 1.1

## Supplementary Materials

The spectra of compounds (**1**, **3**, and **4**) are available online.
