# Supplementary material for: Cytotoxic Stilbenes and Canthinone Alkaloids from Brucea antidysenterica (Simaroubaceae)
Source: Molecules. 2019 Dec 3;24(23):4412. doi: 10.3390/molecules24234412 (PMC6930556; doi:10.3390/molecules24234412)
Supplement: Supplementary file 1 [file molecules-24-04412-s001.pdf]

COMPOUND 1

Figures	Spectra
Figure S1	(+) ESIMS
Figure S2	(-) ESIMS
Figure S3	^{1}H NMR
Figure S4	^{13}C NMR
Figure S5	DEPT
Figure S6	HSQC
Figure S7	HMBC
Figure S8	COSY ^{1}H-^{1}H

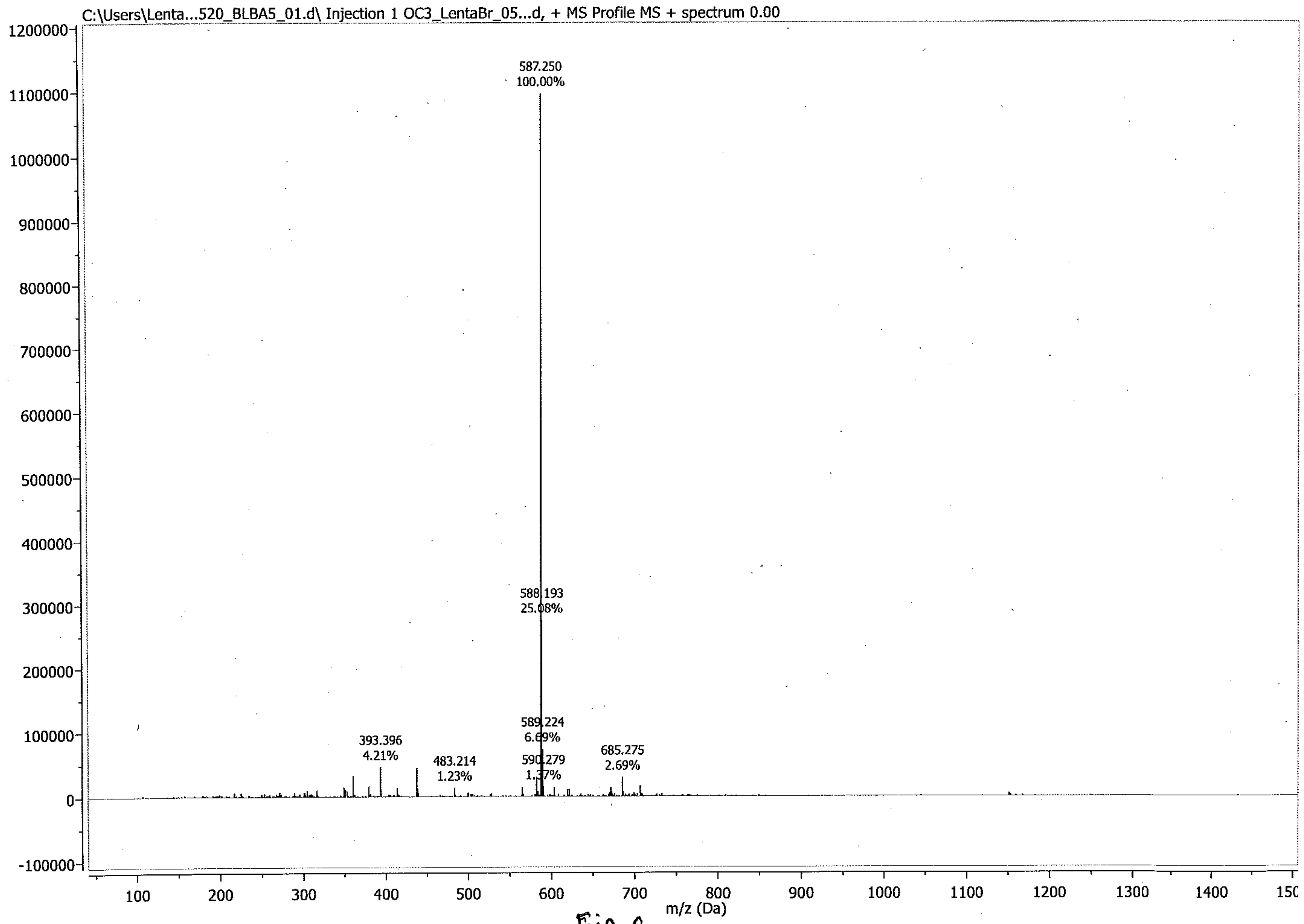
C:\Users\Lenta...520_BLBA5_01.d\ Injection 1 OC3_LentaBr_05...d, + MS Profile MS + spectrum 0.00
1200000
1100000
1000000
900000
800000
700000
600000
500000
400000
300000
200000
100000
0
-100000
587.250
100.00%
588.193
25.08%
589.224
6.69%
590.279
1.37%
393.396
4.21%
483.214
1.23%
685.275
2.69%
100
200
300
400
500
600
700
800
900
1000
1100
1200
1300
1400
150
m/z (Da)

Fig. S1

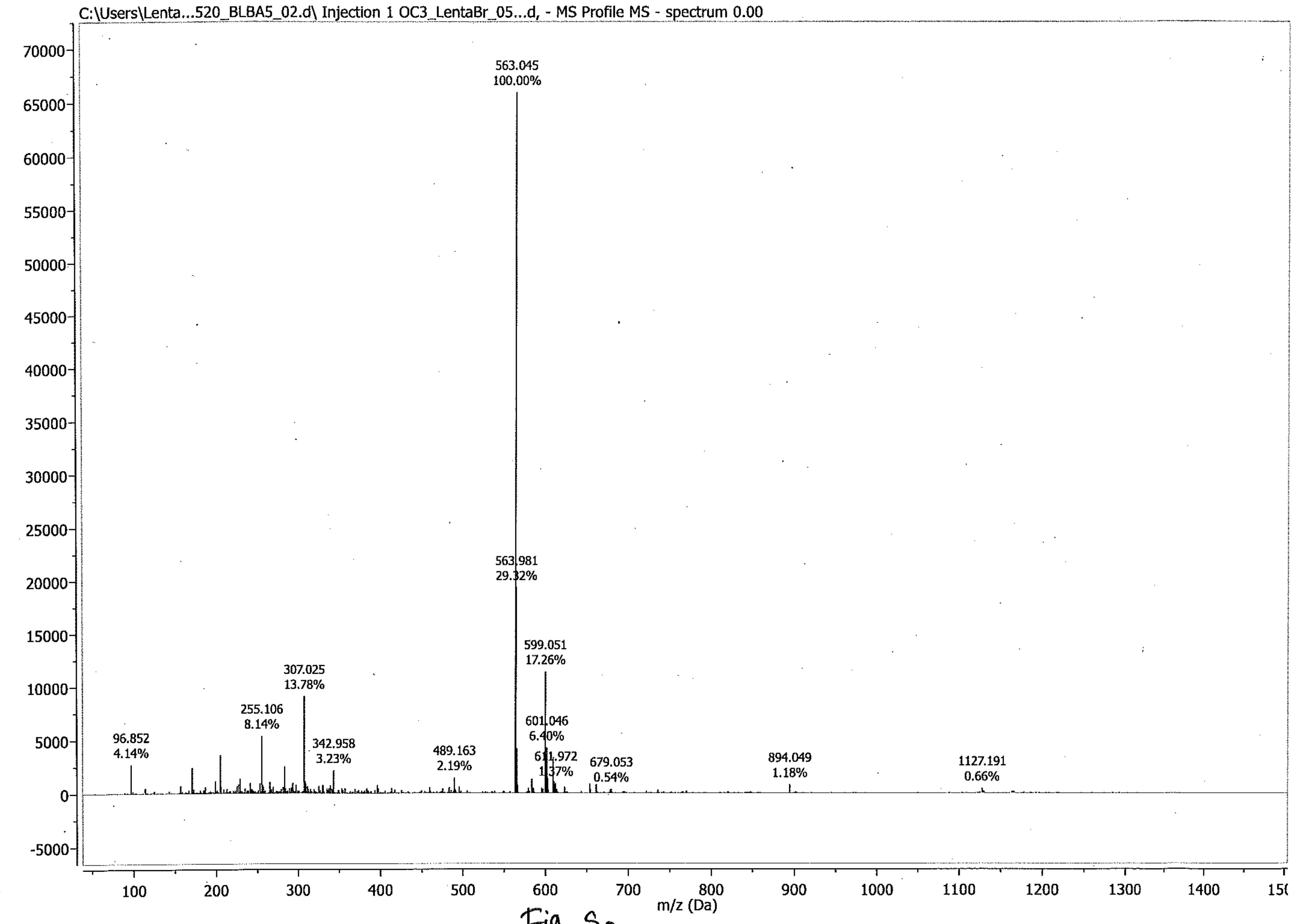
C:\Users\Lenta...520_BLBA5_02.d\ Injection 1 OC3_LentaBr_05...d, - MS Profile MS - spectrum 0.00
70000
65000
60000
55000
50000
45000
40000
35000
30000
25000
20000
15000
10000
5000
0
-5000
563.045 100.00%
563.981 29.32%
599.051 17.26%
307.025 13.78%
255.106 8.14%
601.046 6.40%
96.852 4.14%
342.958 3.23%
489.163 2.19%
611.972 1.37%
679.053 0.54%
894.049 1.18%
1127.191 0.66%
100
200
300
400
500
600
700
800
900
1000
1100
1200
1300
1400
m/z (Da)

Fig. S2

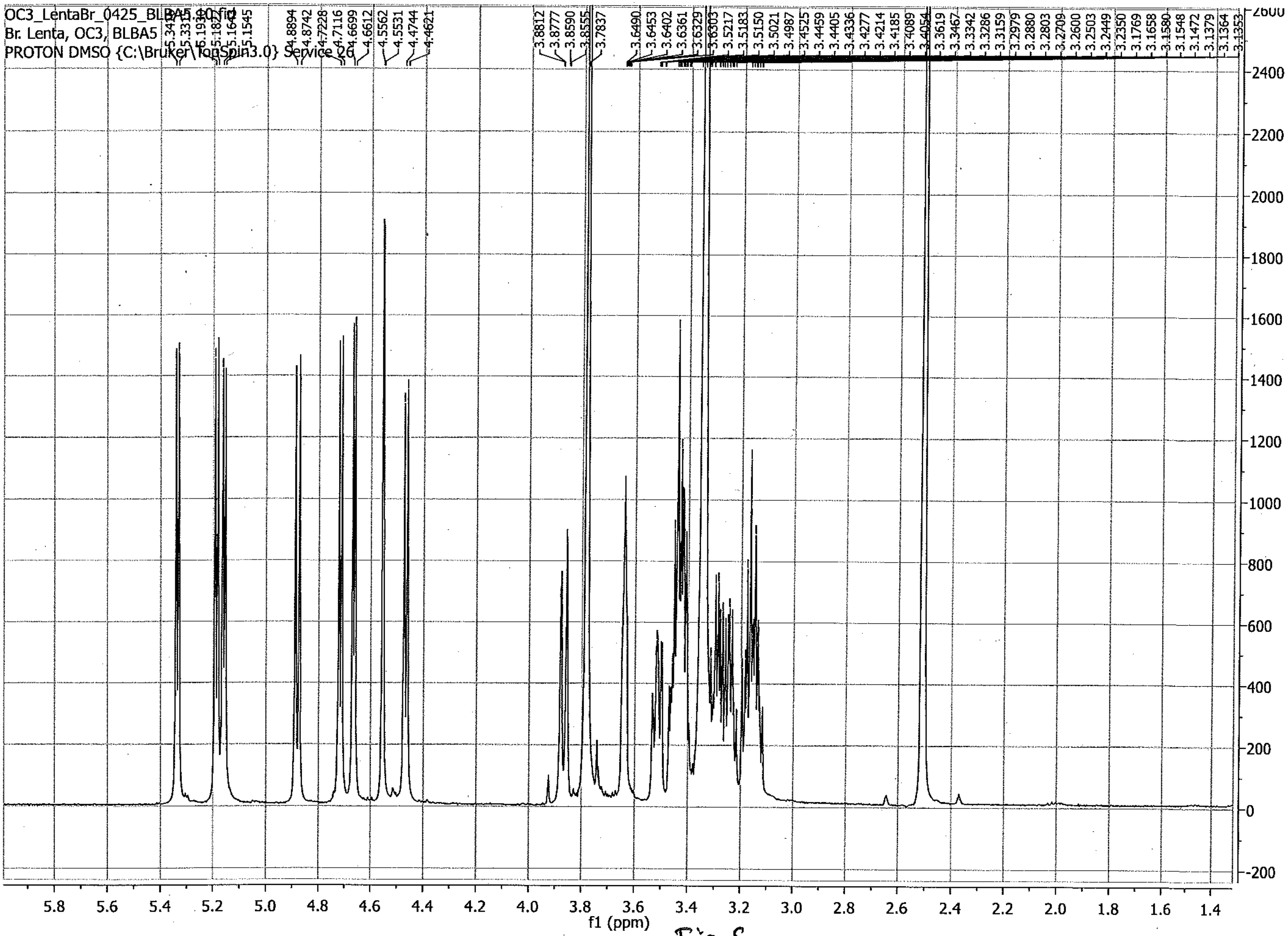
OC3_LentaBr_0425_BLBA5.10.fid
Br. Lenta, OC3, BLBA5
PROTON DMSO {C:\Bruker\TopSpin3.0} Service 26
f1 (ppm)

Fig S8

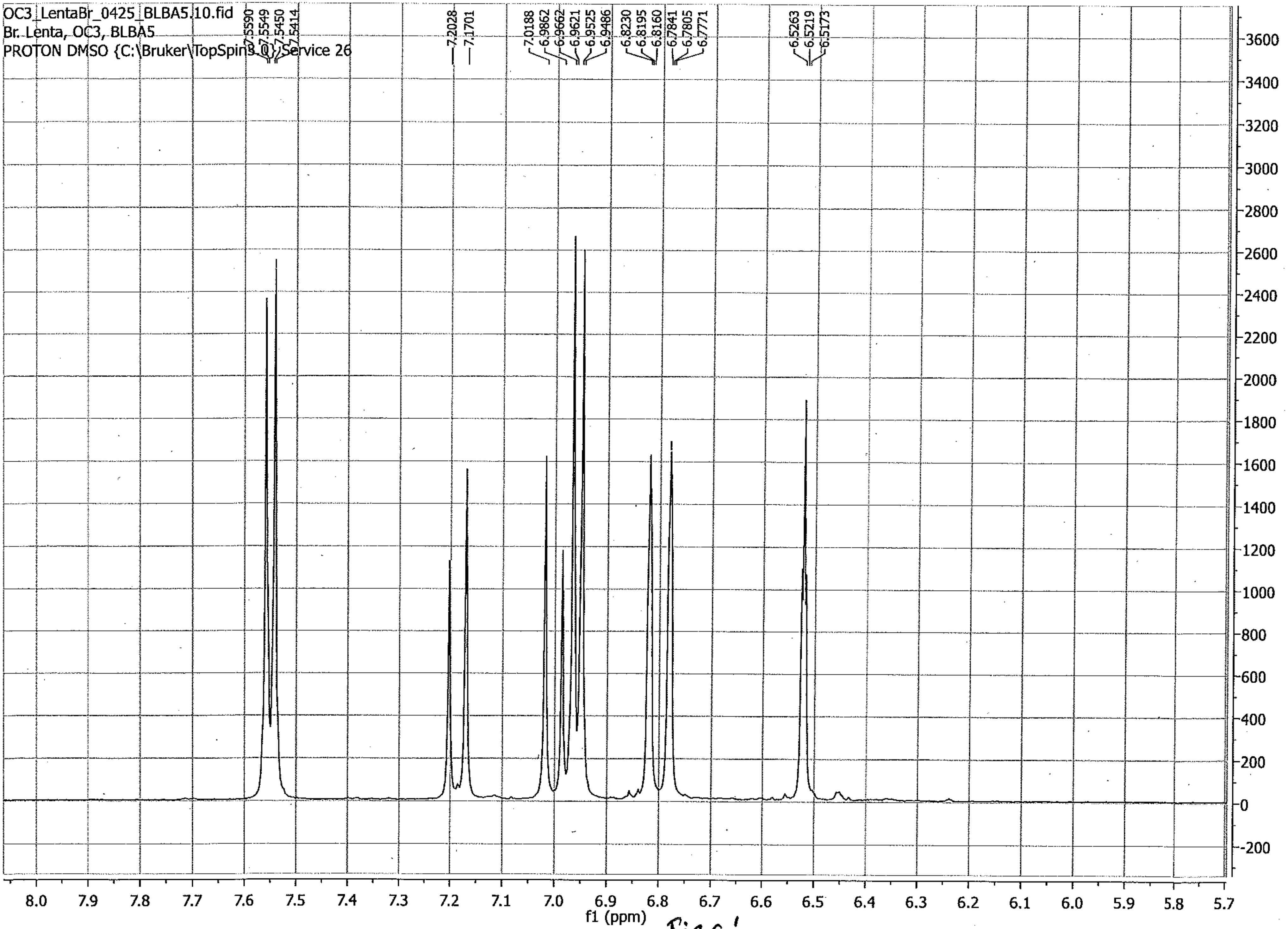
OC3_LentaBr_0425_BLBA5.10.fid
Br. Lenta, OC3, BLBA5
PROTON DMSO {C:\Bruker\TopSpin3.0} Service 26
7.5590
7.5549
7.5450
7.5414
7.2028
7.1701
7.0188
6.9862
6.9662
6.9621
6.9525
6.9486
6.8230
6.8195
6.8160
6.7841
6.7805
6.7771
6.5263
6.5219
6.5173
f1 (ppm)

Fig S3'

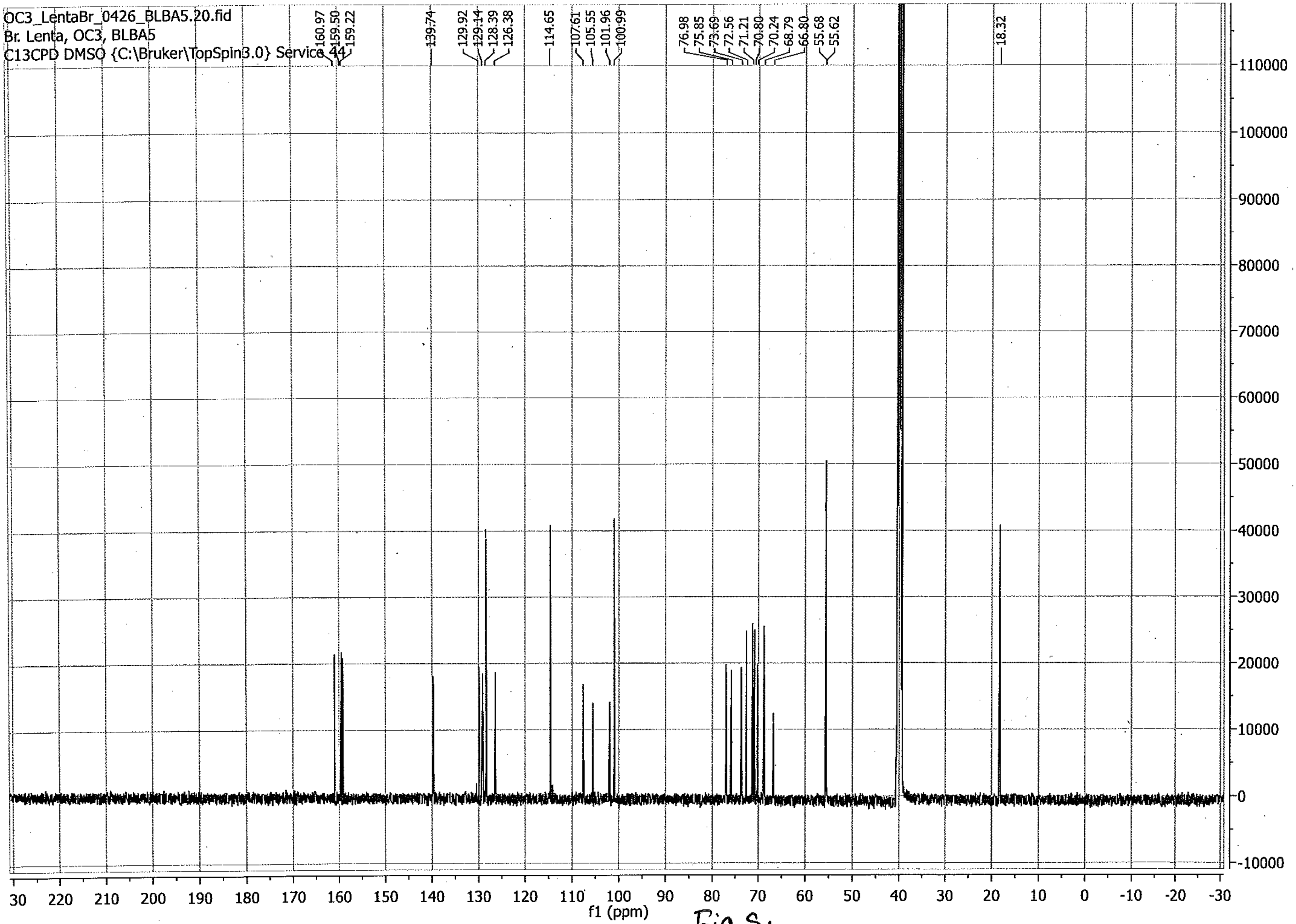
OC3_LentaBr_0426_BLBA5.20.fid
Br. Lenta, OC3, BLBA5
C13CPD DMSO {C:\Bruker\TopSpin3.0} Service 44
160.97
159.50
159.22
139.74
129.92
129.14
128.39
126.38
114.65
107.61
105.55
101.96
100.99
76.98
75.85
73.69
72.56
71.21
70.80
70.24
68.79
66.80
55.68
55.62
18.32
f1 (ppm)
Fig S4

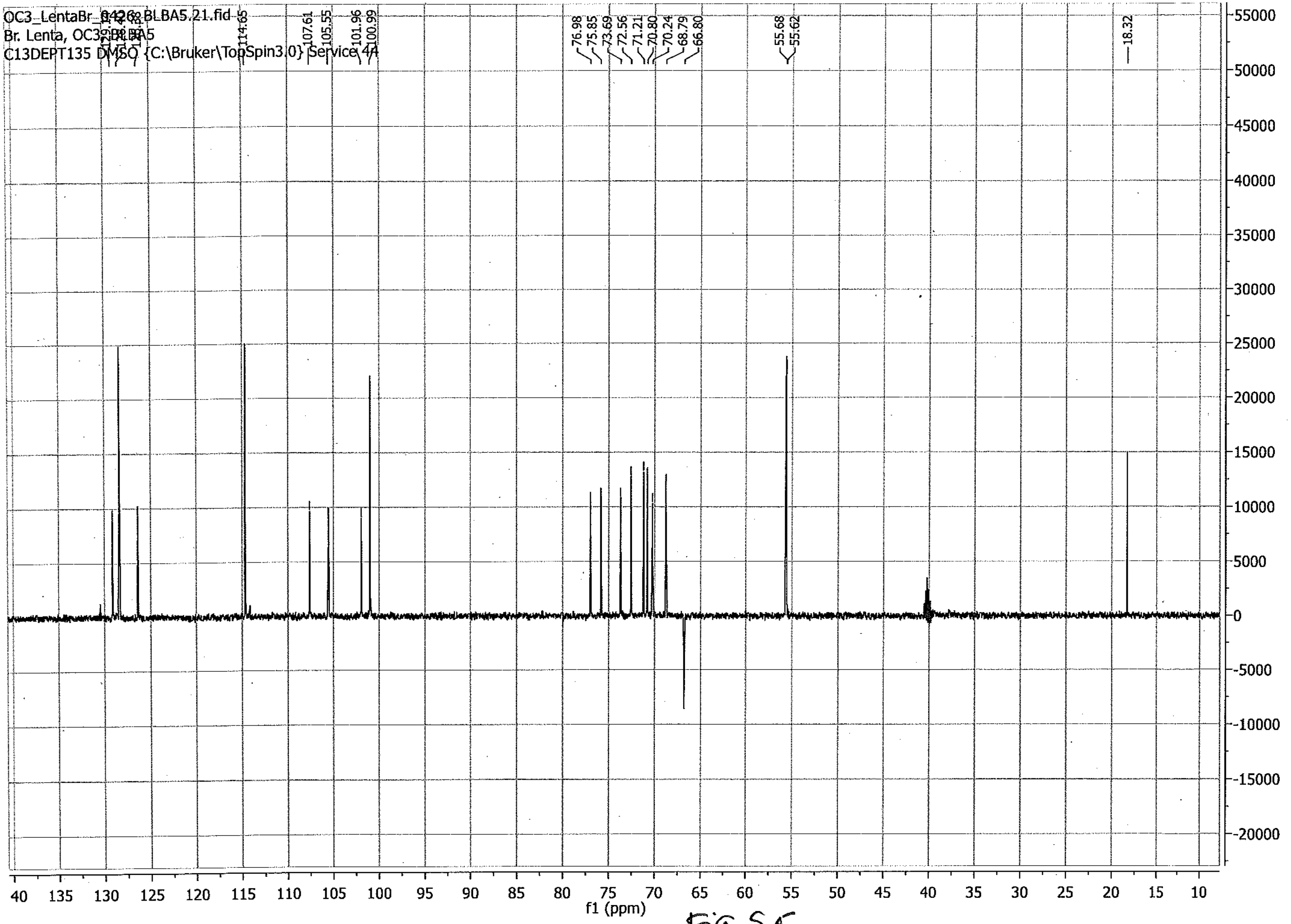
OC3_LentaBr_ ... BLBA5.21.fid
Br. Lenta, OC3 ... 5
C13DEPT135 DMSO {C:\Bruker\TopSpin3.0} Service 44
114.65
107.61
105.55
101.96
100.99
76.98
75.85
73.69
72.56
71.21
70.80
70.24
68.79
66.80
55.68
55.62
18.32
f1 (ppm)

Fig S5

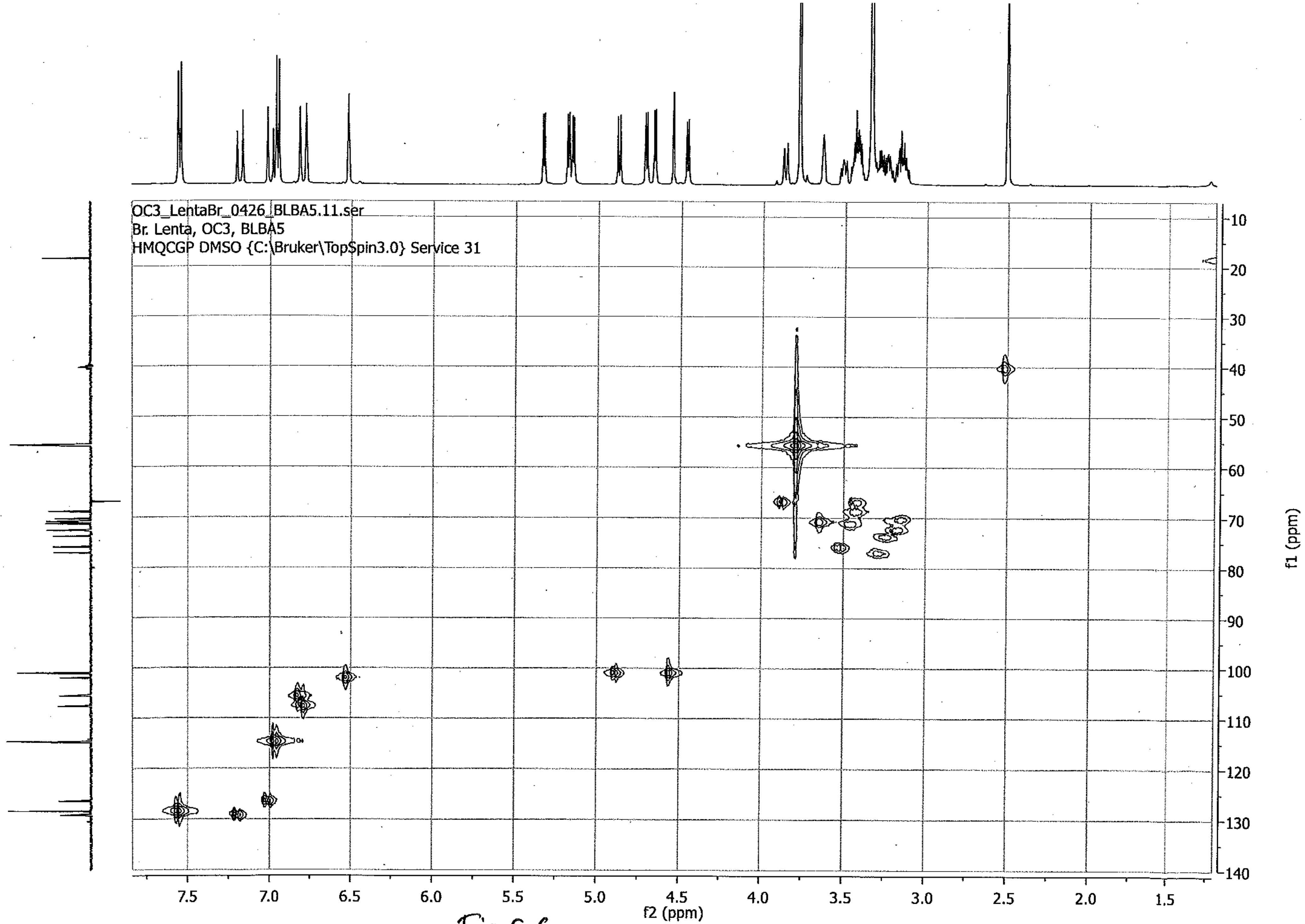
OC3_LentaBr_0426_BLBA5.11.ser
Br. Lenta, OC3, BLBA5
HMQCGP DMSO {C:\Bruker\TopSpin3.0} Service 31
f2 (ppm)
f1 (ppm)
7.5
7.0
6.5
6.0
5.5
5.0
4.5
4.0
3.5
3.0
2.5
2.0
1.5
10
20
30
40
50
60
70
80
90
100
110
120
130
140

Fig 56

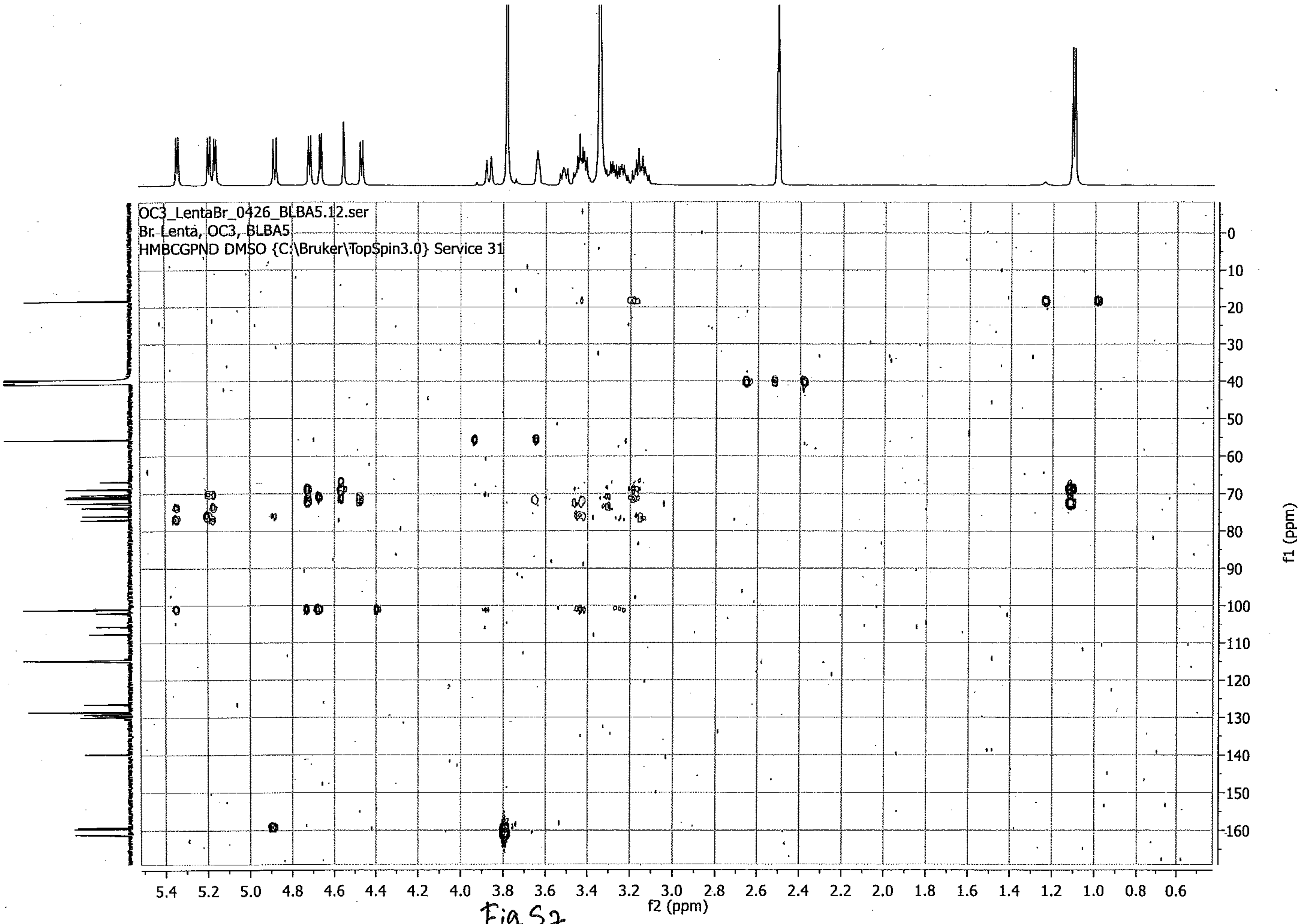
OC3_LentaBr_0426_BLBA5.12.ser
Br. Lenta, OC3, BLBA5
HMBCGPND DMSO {C:\Bruker\TopSpin3.0} Service 31
f2 (ppm)
f1 (ppm)

Fig S7

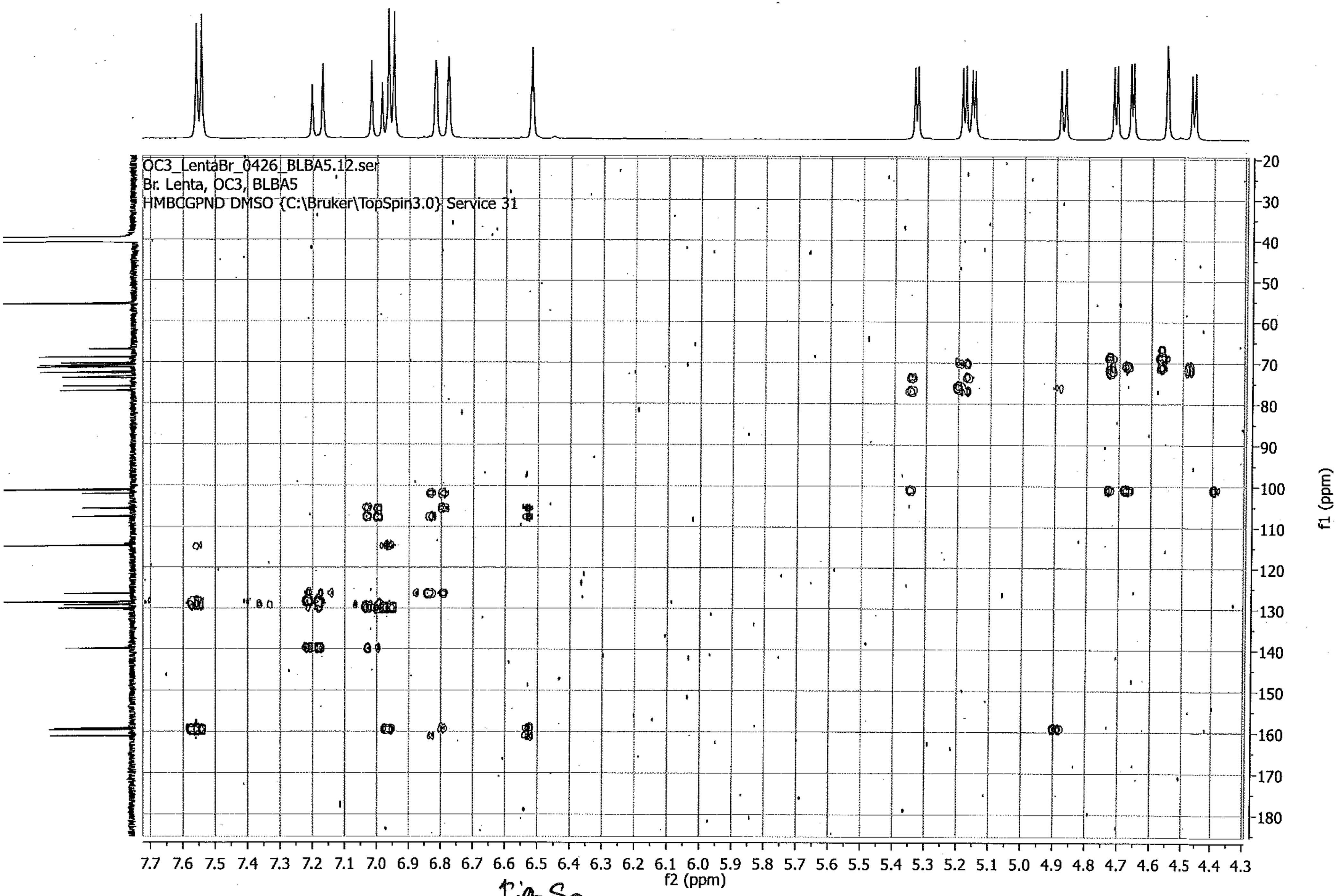
OC3_LentaBr_0426_BLBA5.12.ser
Br. Lenta, OC3, BLBA5
HMBCGPND DMSO {C:\Bruker\TopSpin3.0} Service 31
f2 (ppm)
f1 (ppm)

Fig S2

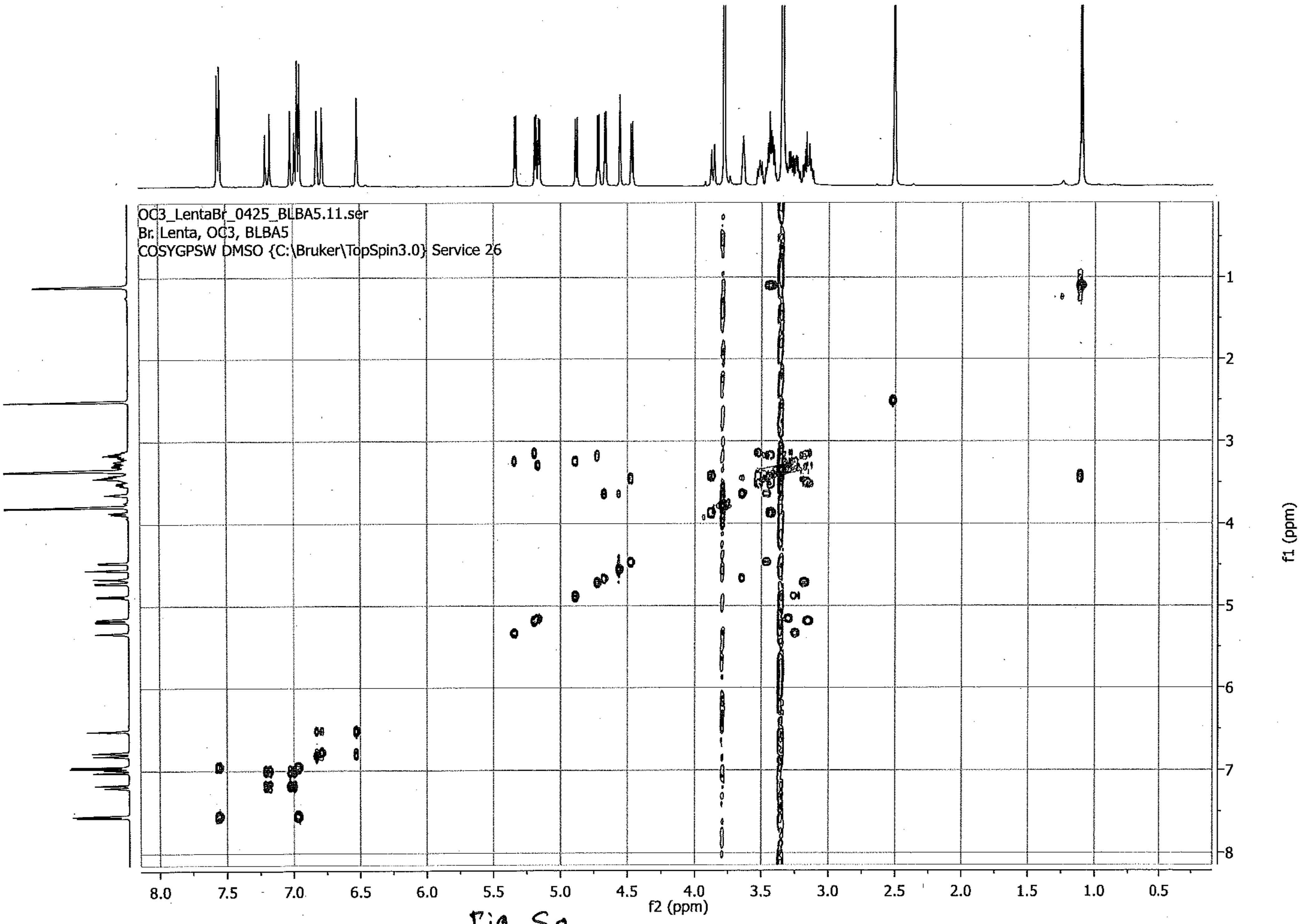
OC3_LentaBr_0425_BLBA5.11.ser
Br. Lenta, OC3, BLBA5
COSYGPSW DMSO {C:\Bruker\TopSpin3.0} Service 26
f2 (ppm)
f1 (ppm)

Fig S8

COMPOUND 3

Figures	Spectra
Figure S9	(+) ESIMS
Figure S10	^{1}H NMR
Figure S11	^{13}C NMR
Figure S12	HSQC
Figure S13	HMBC
Figure S14	COSY ^{1}H-^{1}H

Analysis Report Spectrum

Analysis Info

Analysis Name	OC3_LannangAl_1024
Method	Tune-nan.MS
Workgroup	3OC
Comment	Al. Lannang, OC3

Esquire 3000

Universität Bielefeld

Operator	S.Heitkamp
Acquisition Date	28.10.2019 10:17:48
Print Date	28.10.2019 11:43:28

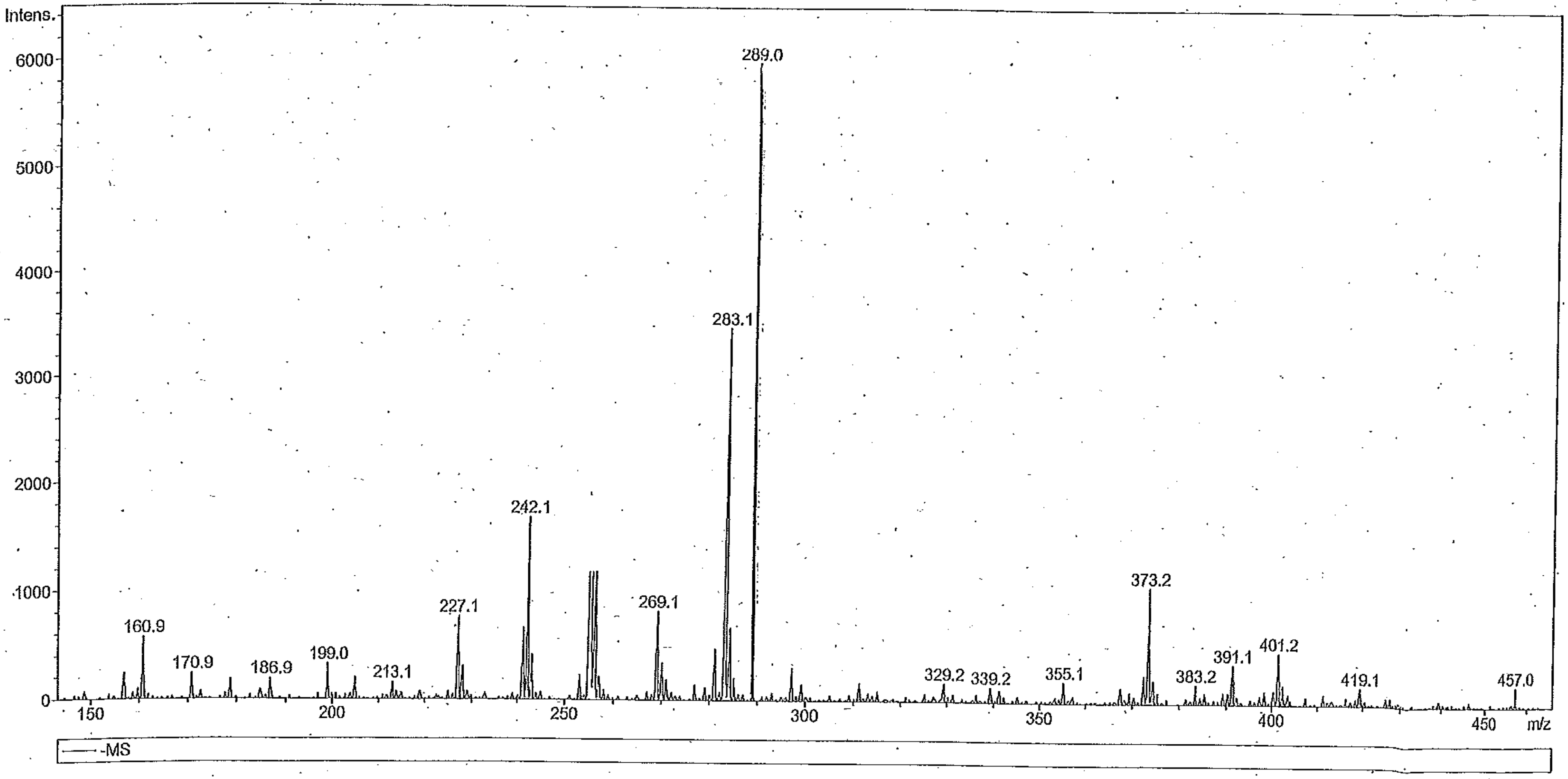

Fig S9

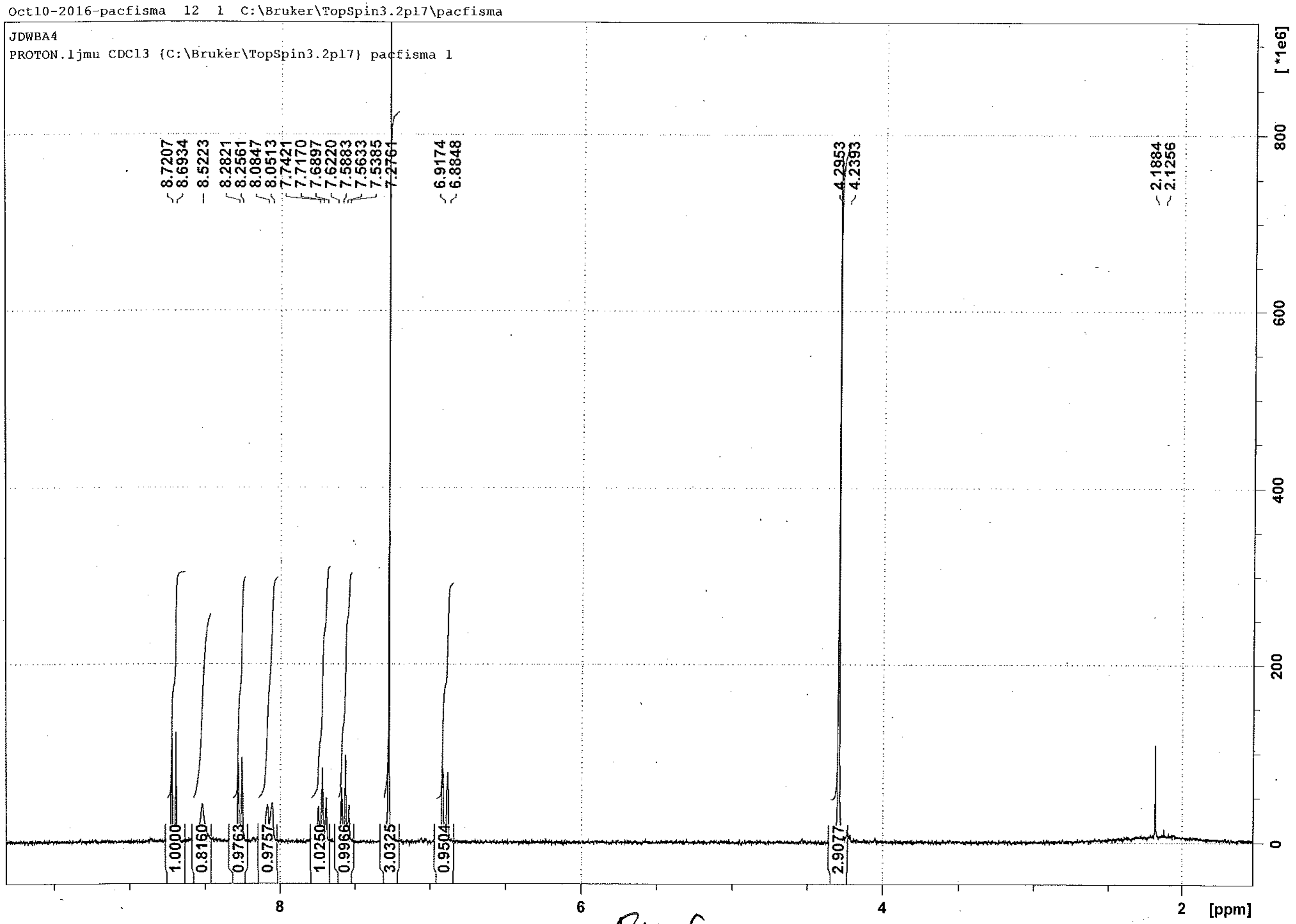
Oct10-2016-pacfisma 12 1 C:\Bruker\TopSpin3.2pl7\pacfisma
JDWBA4
PROTON.ljmu CDCl3 {C:\Bruker\TopSpin3.2pl7} pacfisma 1
8.7207
8.6934
8.5223
8.2821
8.2561
8.0847
8.0513
7.7421
7.7170
7.6897
7.6220
7.5883
7.5633
7.5385
7.2761
6.9174
6.8848
4.2953
4.2393
2.1884
2.1256
1.0000
0.8160
0.9763
0.9757
1.0250
0.9966
3.0325
0.9504
2.9077
[*1e6]
800
600
400
200
0
8
6
4
2
[ppm]

Fig S10

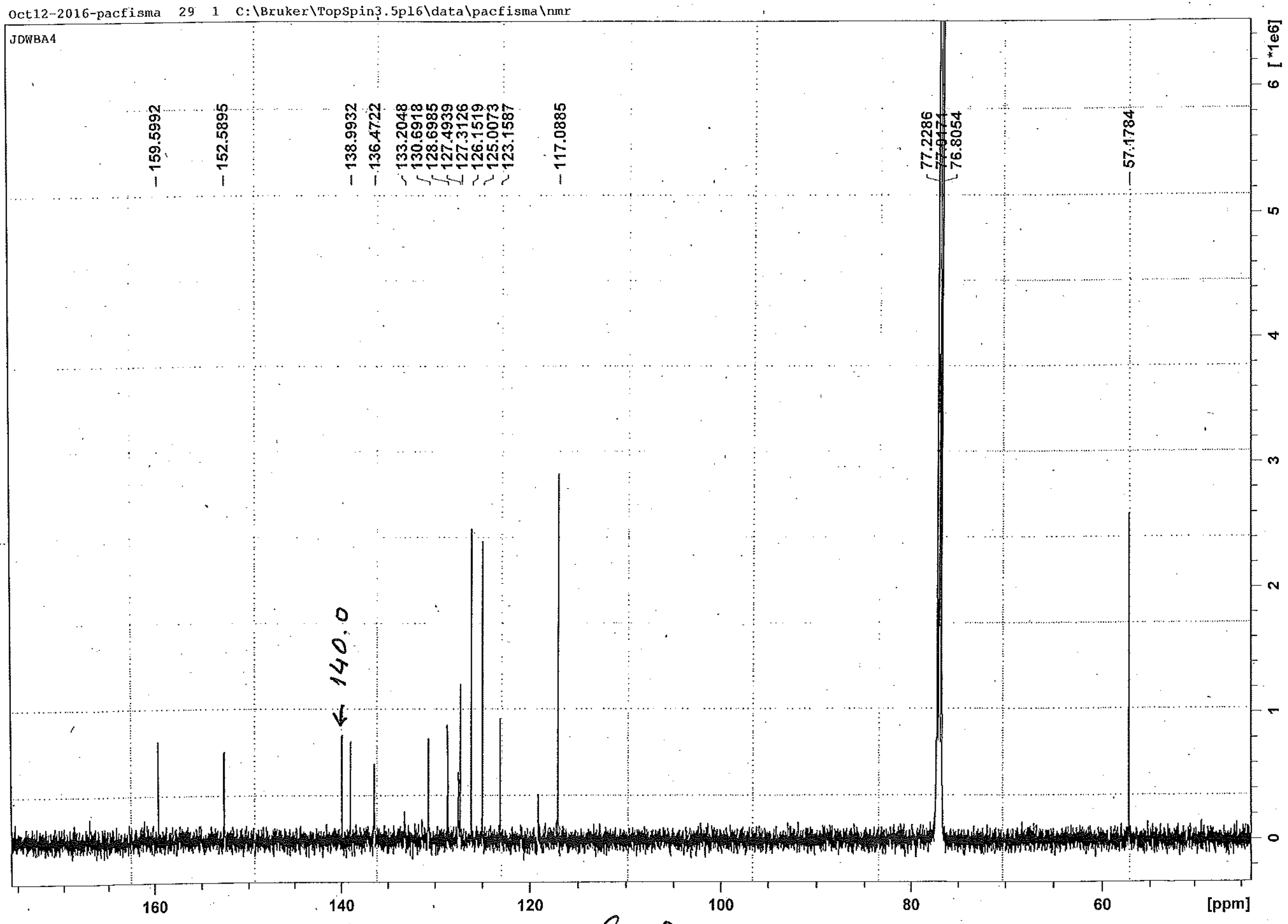
Oct12-2016-pacfisma 29 1 C:\Bruker\TopSpin3.5pl6\data\pacfisma\nmr
JDWBA4
159.5992
152.5895
138.9932
136.4722
133.2048
130.6918
128.6985
127.4939
127.3126
126.1519
125.0073
123.1587
117.0885
77.2286
76.8054
57.1784
140.0
160
140
120
100
80
60
[ppm]
0
1
2
3
4
5
6
[*1e6]

Fig S11

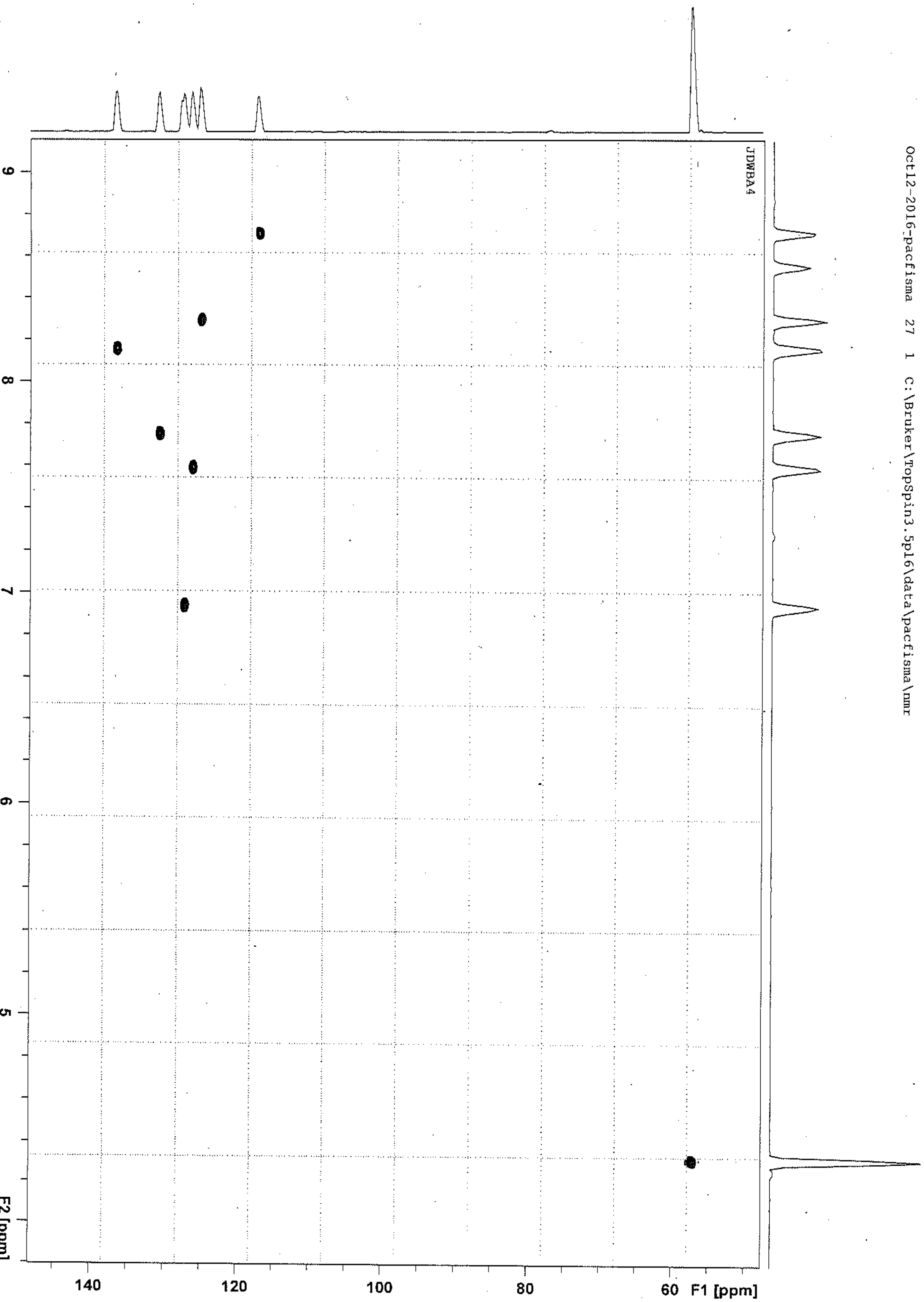

Oct12-2016_pacfisma 27 1 C:\Bruker\TopSpin3.5pl6\data\pacfisma\nmr
JDWBA4
F2 [ppm]
9
8
7
6
5
F1 [ppm]
140
120
100
80
60

Fig S12

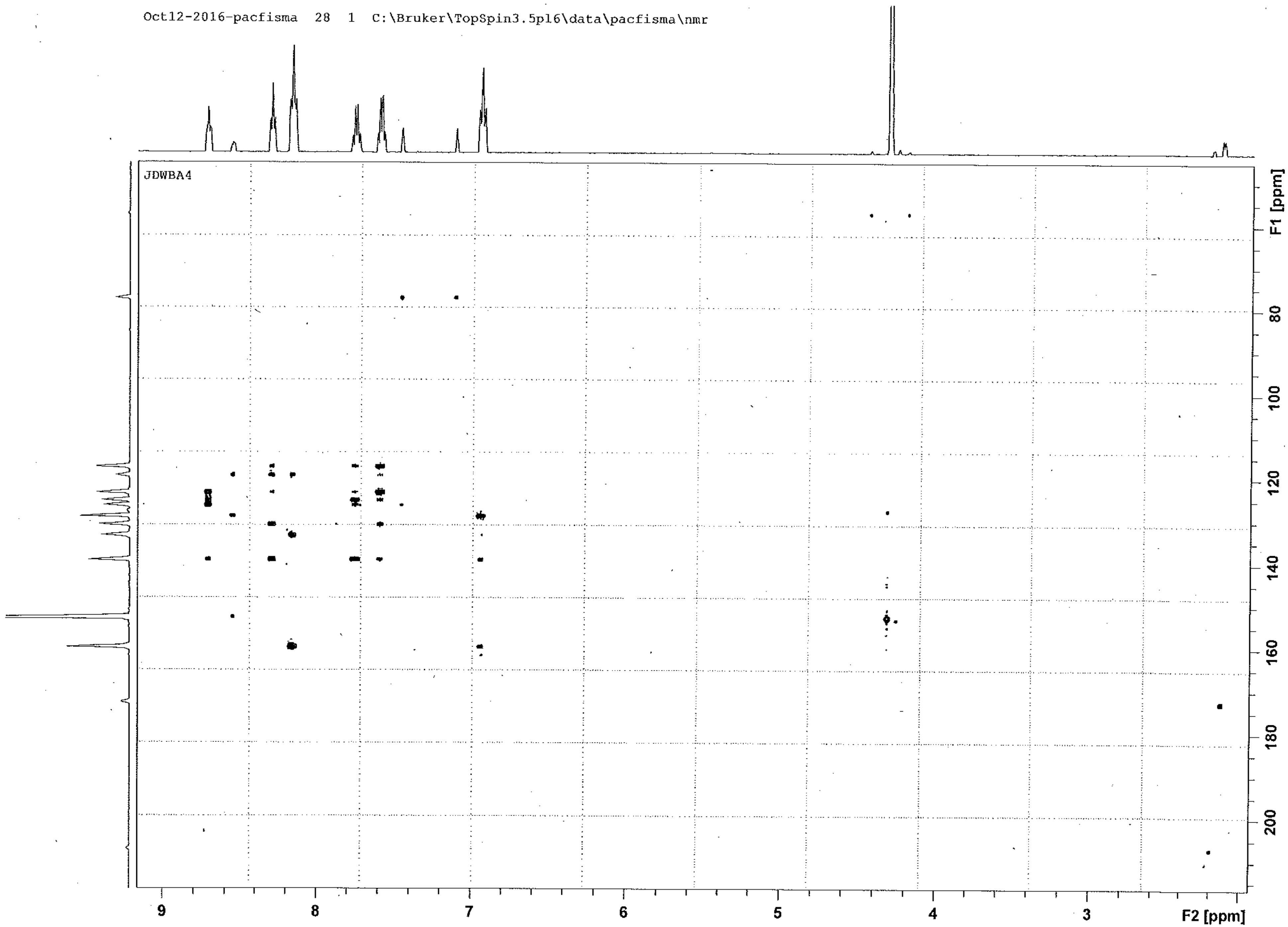
Oct12-2016-pacfisma 28 1 C:\Bruker\TopSpin3.5pl6\data\pacfisma\nmr
JDWBA4
F1 [ppm]
80
100
120
140
160
180
200
9
8
7
6
5
4
3
F2 [ppm]

Fig S13

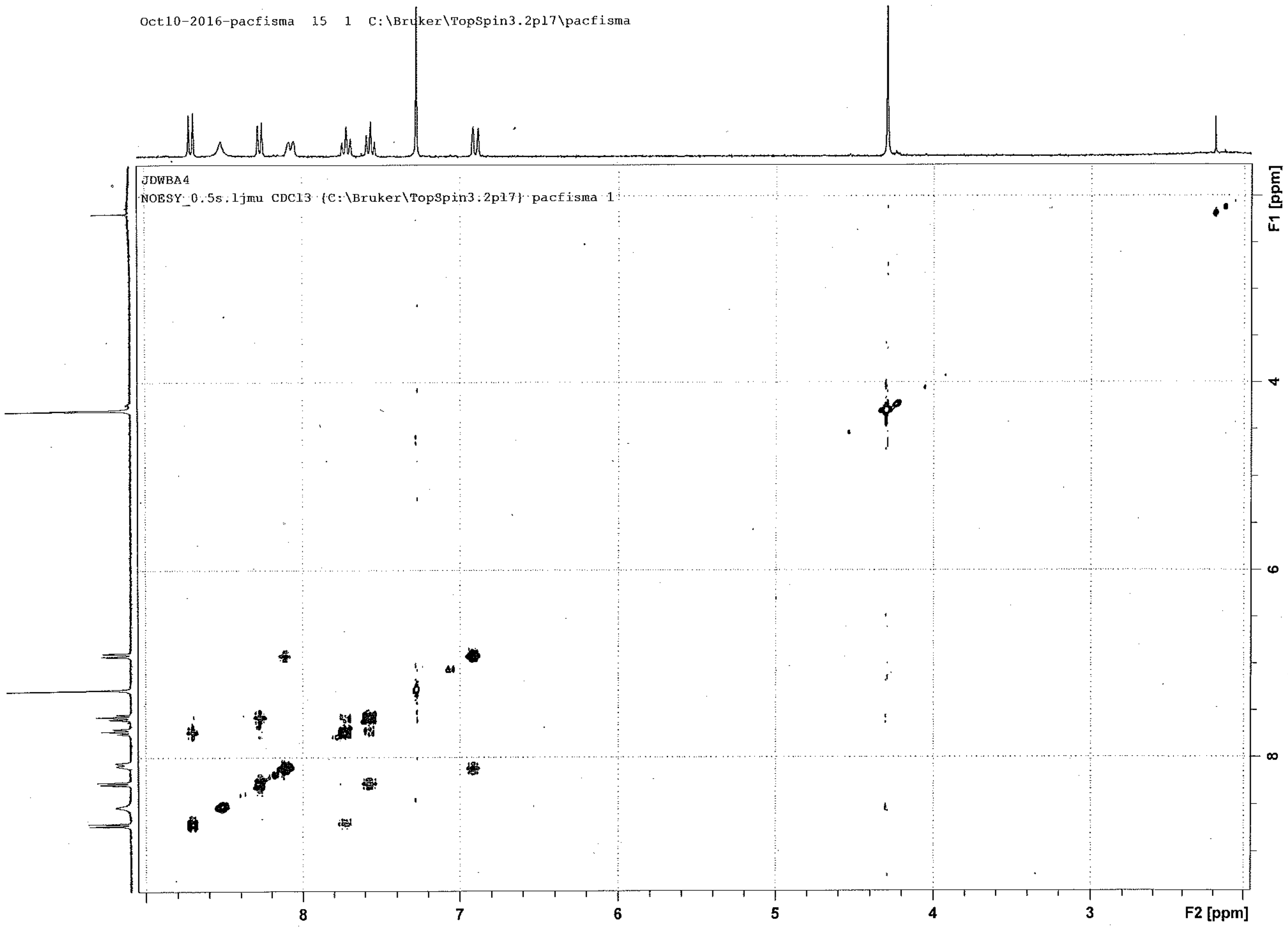
Oct10-2016-pacfisma 15 1 C:\Bruker\TopSpin3.2pl7\pacfisma
JDWBA4
NOESY_0.5s.ljmu CDCl3 {C:\Bruker\TopSpin3.2pl7} pacfisma 1
F1 [ppm]
F2 [ppm]
8
7
6
5
4
3
4
6
8

Fig S14

COMPOUND 4

Figures	Spectra
Figure S15	(+) ESIMS
Figure S16	^{1}H NMR
Figure S17	^{13}C NMR
Figure S18	HMBC
Figure S19	HSQC
Figure S20	COSY ^{1}H-^{1}H

Analysis Report Spectrum

Analysis Info

Analysis Name	OC3_Tadjong-TchoAl_1002
Method	Tune-nan.MS
Workgroup	3OC
Comment	Al, Tadjong-Tcho, OC3

Esquire 3000

Universität Bielefeld

Operator	S.Heitkamp
Acquisition Date	09.10.2019 11:17:08
Print Date	09.10.2019 12:17:30

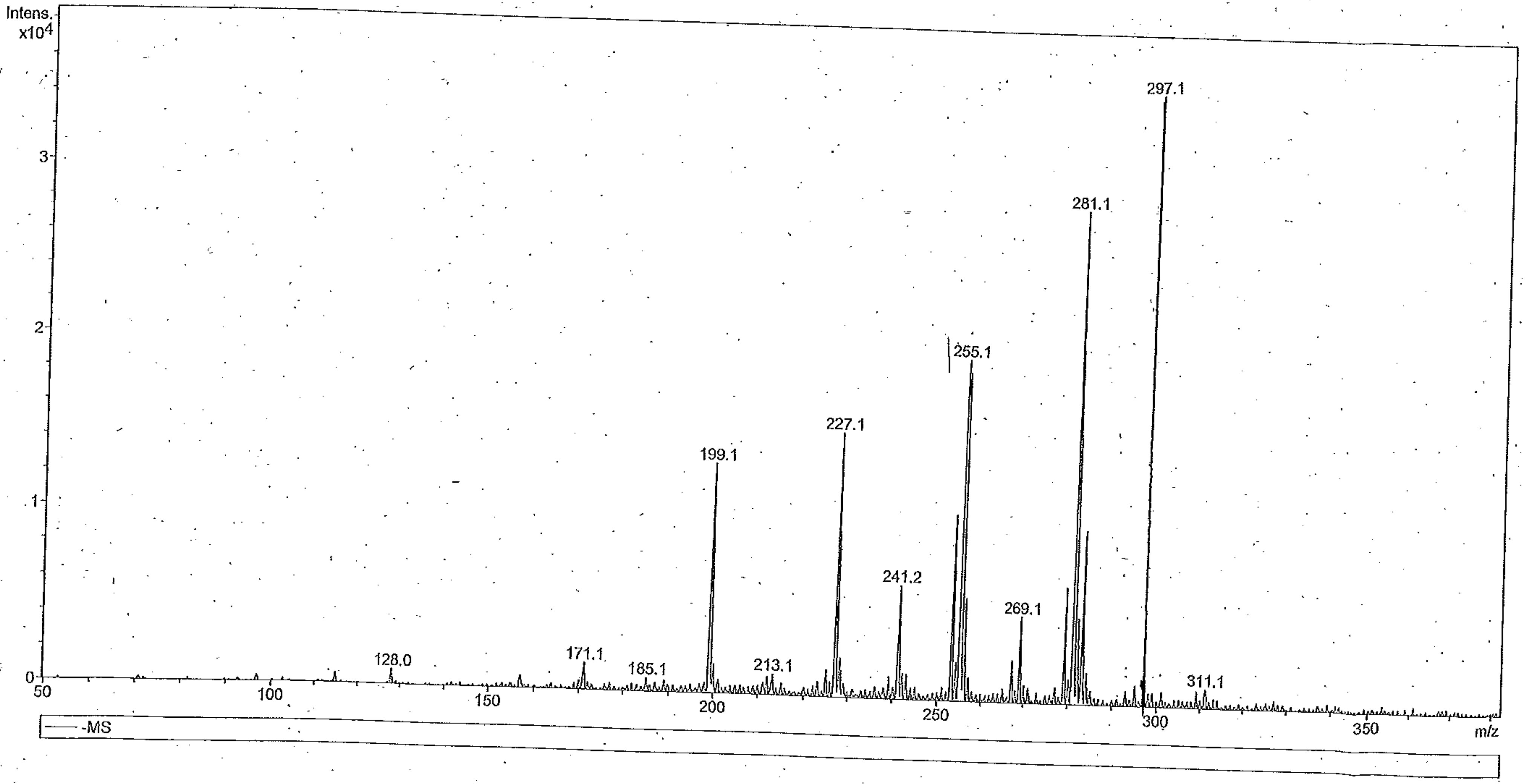

Fig S15

Fig 3/16

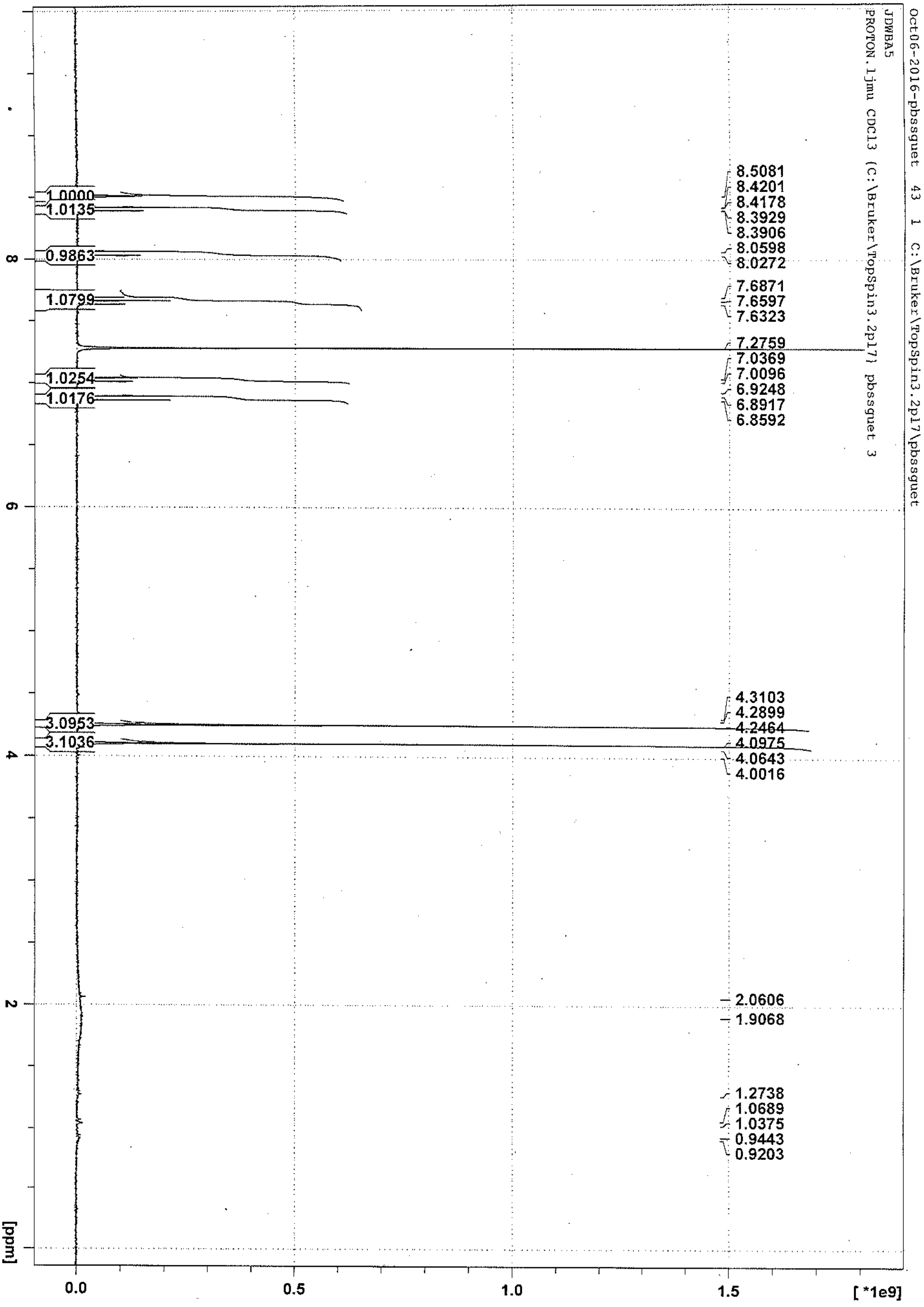

Oct06-2016-pbssguet 43 1 C:\Bruker\TopSpin3.2pl7\pbssguet
JDWBA5
PROTON.1jmu CDCl3 {C:\Bruker\TopSpin3.2pl7} pbssguet 3
8.5081
8.4201
8.4178
8.3929
8.3906
8.0598
8.0272
7.6871
7.6597
7.6323
7.2759
7.0369
7.0096
6.9248
6.8917
6.8592
4.3103
4.2899
4.2464
4.0975
4.0643
4.0016
2.0606
1.9068
1.2738
1.0689
1.0375
0.9443
0.9203
1.0000
1.0135
0.9863
1.0799
1.0254
1.0176
3.0953
3.1036
8
6
4
2
[ppm]
0.0
0.5
1.0
1.5
[*1e9]

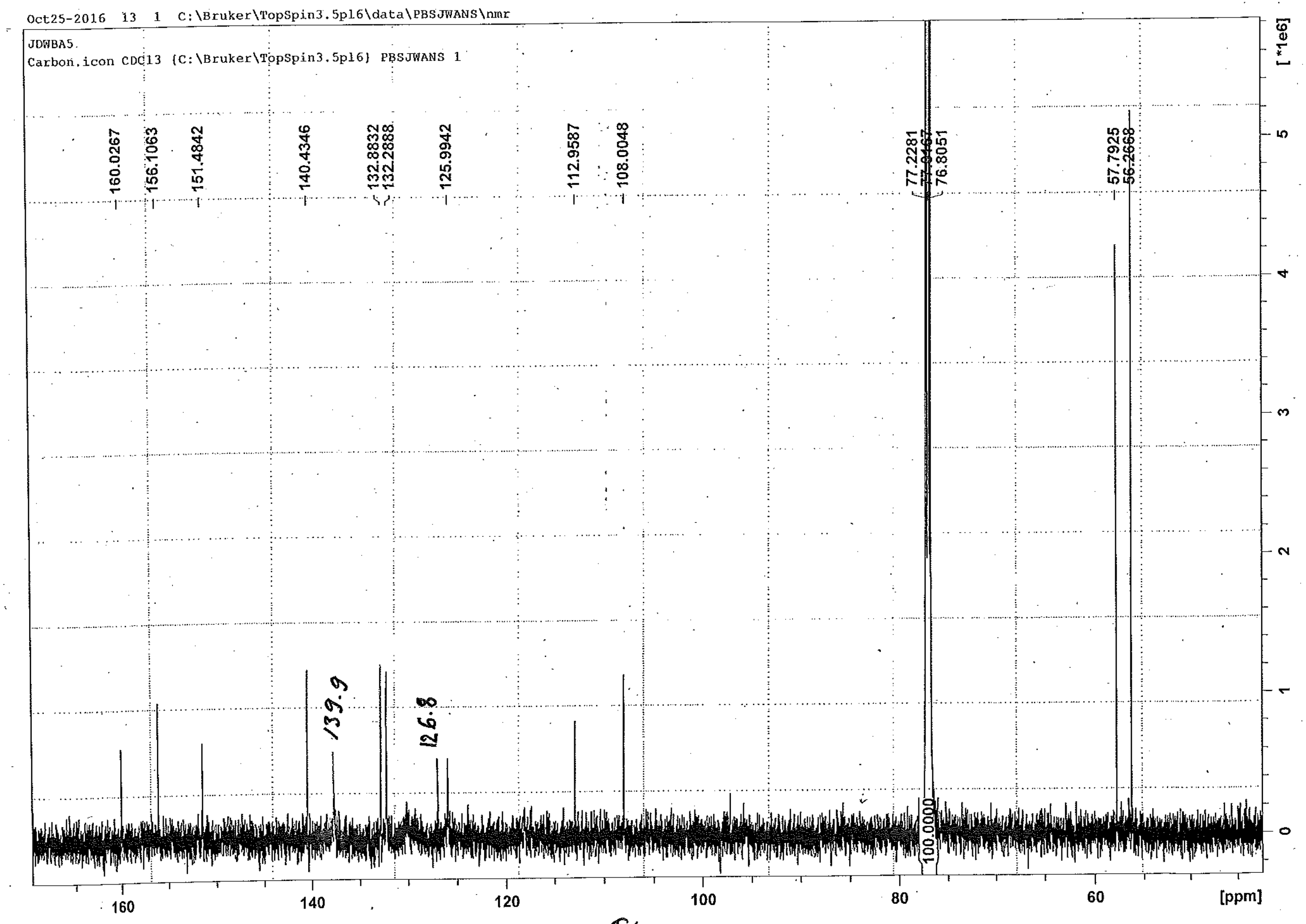
Oct25-2016 13 1 C:\Bruker\TopSpin3.5pl6\data\PBSJWANS\nmr
JDWBA5.
Carbon.icon CDCl3 (C:\Bruker\TopSpin3.5pl6) PBSJWANS 1
160.0267
156.1063
151.4842
140.4346
132.8832
132.2888
125.9942
112.9587
108.0048
77.2281
76.8051
57.7925
56.2668
139.9
126.8
100.0000
160
140
120
100
80
60
[ppm]
0
1
2
3
4
5
[*1e6]

Fig S17

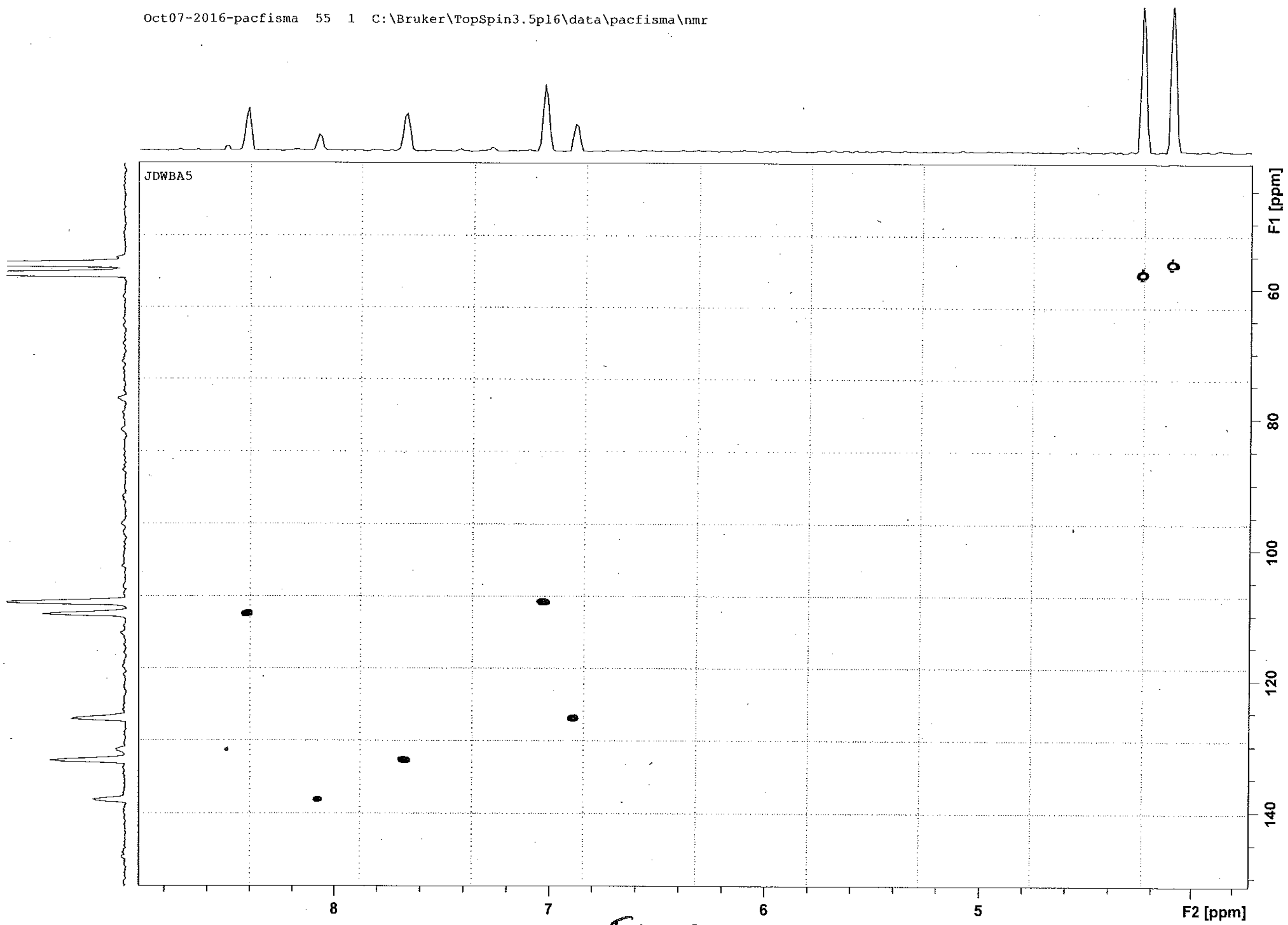
Oct07-2016-pacfisma 55 1 C:\Bruker\TopSpin3.5pl6\data\pacfisma\nmr
JDWBA5
F1 [ppm]
60
80
100
120
140
8
7
6
5
F2 [ppm]

Fig S18

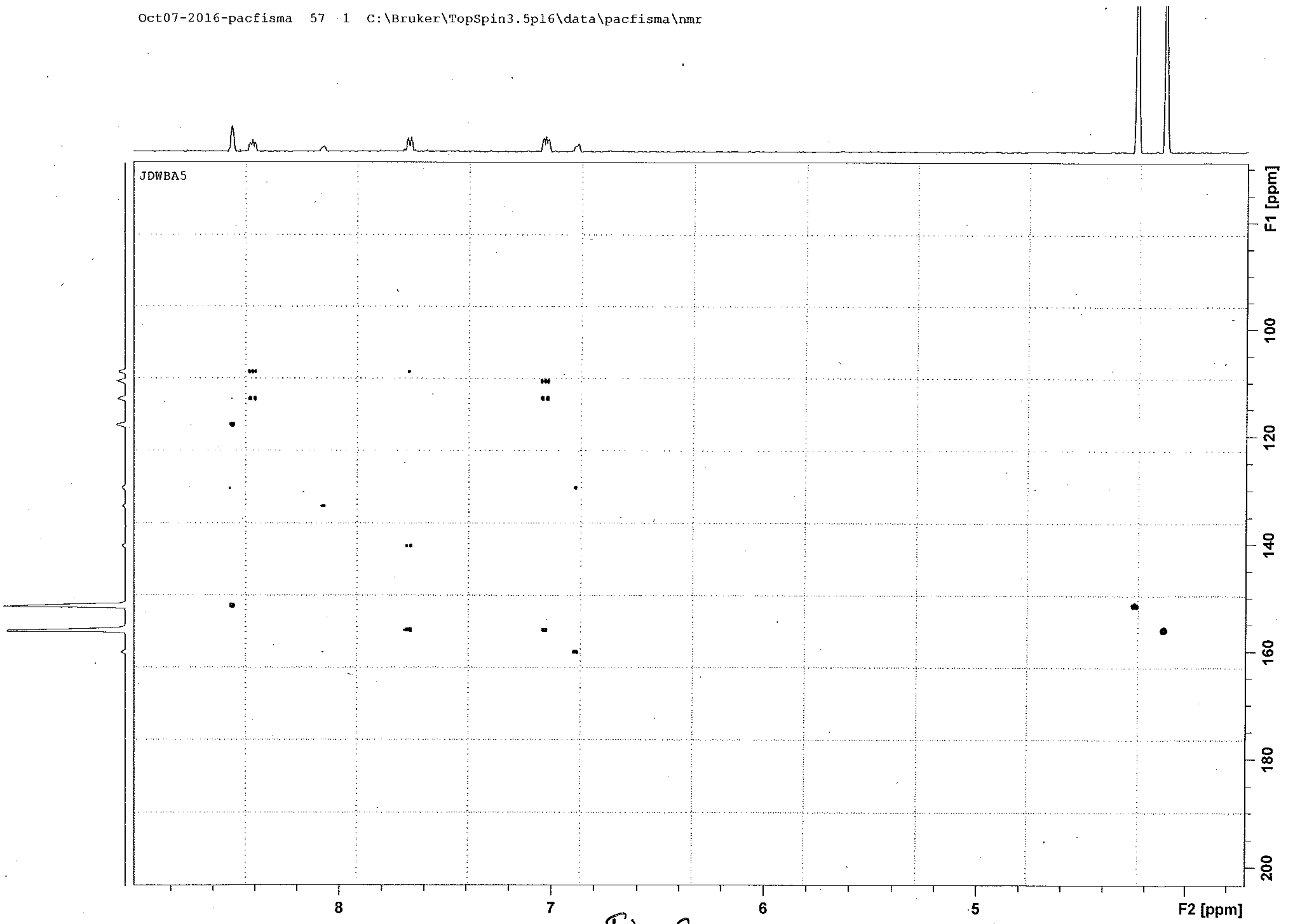
Oct07-2016-pacfisma 57 1 C:\Bruker\TopSpin3.5pl6\data\pacfisma\nmr
JDWBA5
F1 [ppm]
100
120
140
160
180
200
8
7
6
5
F2 [ppm]

Fig S19

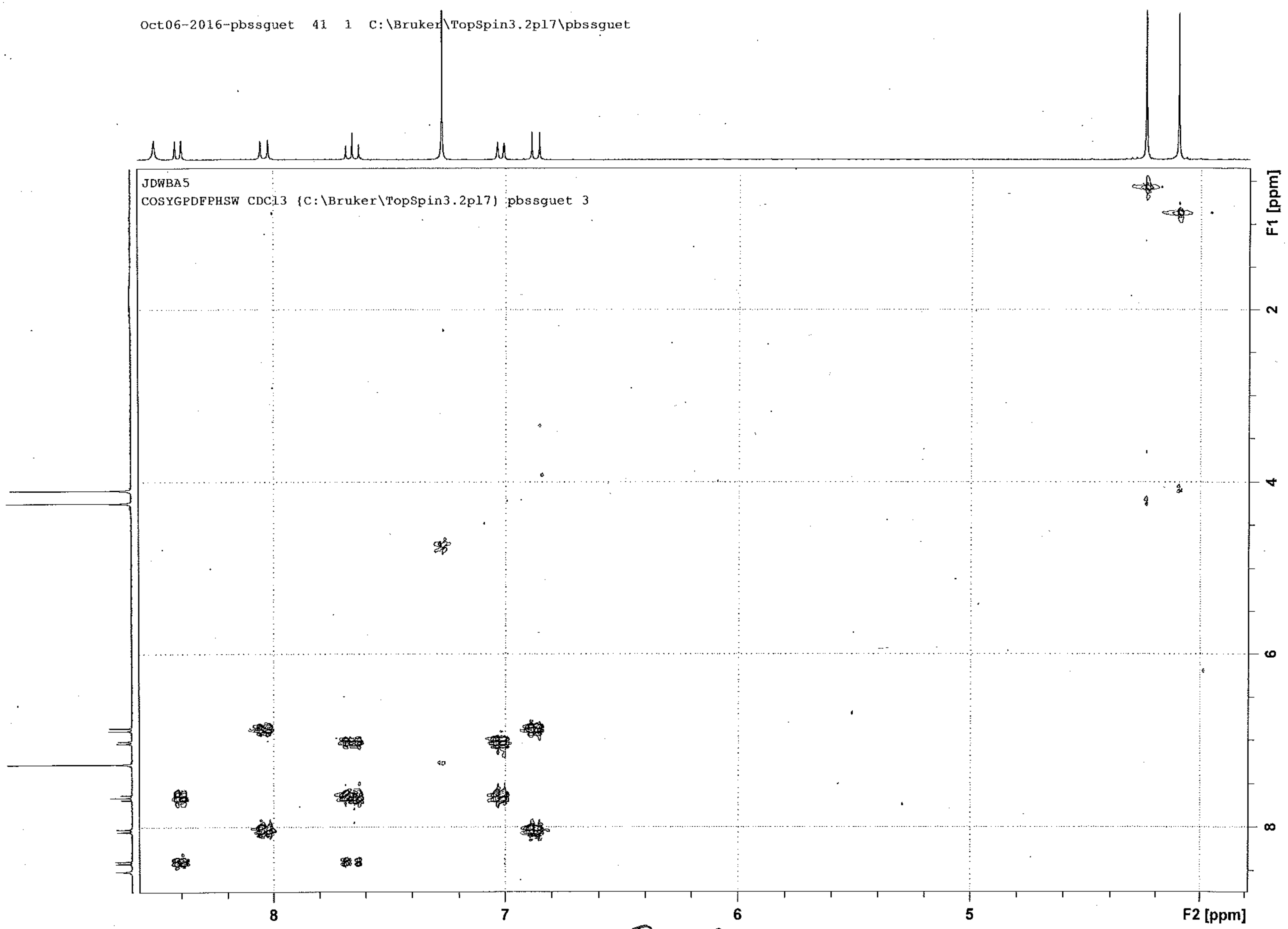
Oct06-2016-pbssguet 41 1 C:\Bruker\TopSpin3.2pl7\pbssguet
JDWBA5
COSYGPDFPHSW CDCl3 {C:\Bruker\TopSpin3.2pl7} pbssguet 3
F1 [ppm]
F2 [ppm]
8
7
6
5
2
4
6
8

Fig S20
